# Unconventionally secreted effectors of two filamentous pathogens target plant salicylate biosynthesis

**DOI:** 10.1038/ncomms5686

**Published:** 2014-08-26

**Authors:** Tingli Liu, Tianqiao Song, Xiong Zhang, Hongbo Yuan, Liming Su, Wanlin Li, Jing Xu, Shiheng Liu, Linlin Chen, Tianzi Chen, Meixiang Zhang, Lichuan Gu, Baolong Zhang, Daolong Dou

**Affiliations:** 1 Department of Plant Pathology, Nanjing Agricultural University, 1 Weigang Road, Nanjing 210095, China; 2 Provincial Key Laboratory of Agrobiology, Jiangsu Academy of Agricultural Sciences, Nanjing 210095, China; 3 State Key Laboratory of Microbial Technology, Shandong University, Jinan 250100, China; 4 These authors contributed equally to this work

## Abstract

Plant diseases caused by fungi and oomycetes pose an increasing threat to food security and ecosystem health worldwide. These filamentous pathogens, while taxonomically distinct, modulate host defense responses by secreting effectors, which are typically identified based on the presence of signal peptides. Here we show that *Phytophthora sojae* and *Verticillium dahliae* secrete isochorismatases (PsIsc1 and VdIsc1, respectively) that are required for full pathogenesis. PsIsc1 and VdIsc1 can suppress salicylate-mediated innate immunity *in planta* and hydrolyse isochorismate *in vitro*. A conserved triad of catalytic residues is essential for both functions. Thus, the two proteins are isochorismatase effectors that disrupt the plant salicylate metabolism pathway by suppressing its precursor. Furthermore, these proteins lack signal peptides, but exhibit characteristics that lead to unconventional secretion. Therefore, this secretion pathway is a novel mechanism for delivering effectors and might play an important role in host–pathogen interactions.

## Abstract (web summary)

Salicylate is a regulator of innate immunity to infection in plants. Here, Liu *et al.* show that two plant pathogens secrete enzymes that disrupt salicylate biosynthesis and plant immunity, and reveal that these effectors are secreted via an unconventional mechanism.

## Graphical abstract

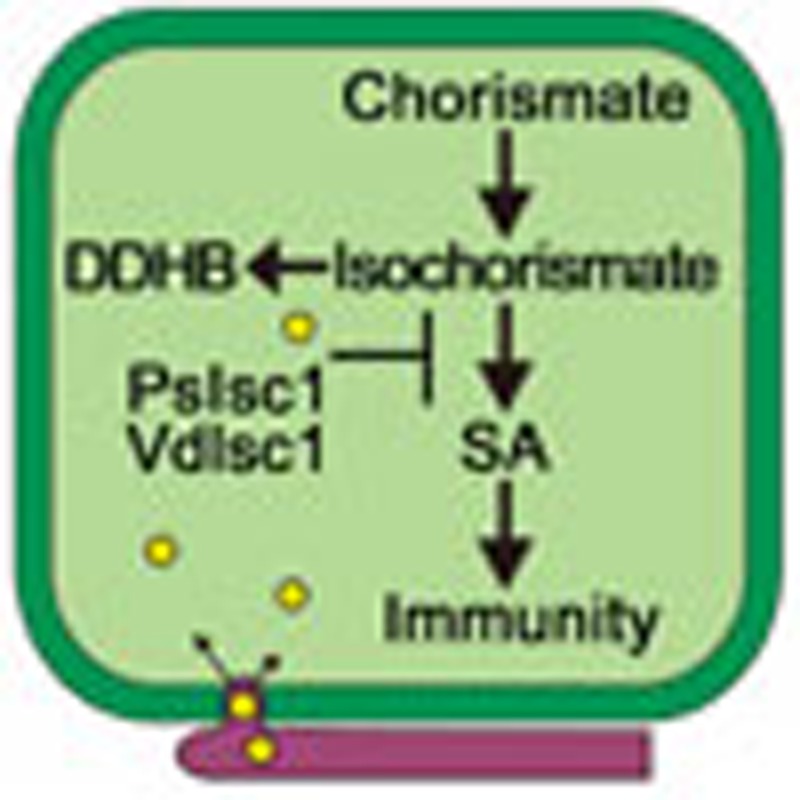

P*hytophthora sojae* is an oomycete pathogen from soybean^1^, whereas *Verticillium dahliae* is a vascular wilt fungus that causes diseases in >200 plant species^2^. Although they are evolutionarily distant eukaryotes, both are filamentous pathogens that grow or infect their hosts via thread-like hyphae. These filamentous plant pathogens secrete a complex repertoire of effector proteins to manipulate host innate immunity^3^,^4^. The effectors can either contribute to pathogen virulence by overcoming plant defense (termed effector-triggered susceptibility), or be directly or indirectly recognized by host surveillance systems, leading to effector-triggered immunity^5^. Typically, the secreted effectors are characterized by short N-terminal signal peptides that direct them to the secretory pathway^3^,^6^. *P. sojae* produces hundreds of apoplastic effectors and putative cell-entering effectors^1^,^3^,^6^, whereas *V. dahliae* secretes more than 700 putative effectors, including many small cysteine-rich proteins that are thought to function in the host apoplast to promote disease^2^. For example, the *Verticillium* effector Ave1 contributes to host fungal virulence in the absence of its corresponding R protein (Ve1)^7^. Nevertheless, the virulence targets and mode of action of these effectors remain largely unknown.

Salicylate (SA) is a central signaling molecule during plant innate immune responses. It is usually induced by infection with biotrophic or hemibiotrophic pathogens, and serves as an important component of PAMP-triggered immunity (PTI), effector-triggered immunity and systemic acquired resistance^8^,^9^. Plants synthesize SA through two distinct enzymatic pathways. One is from phenylalanine via cinnamate and the other is from chorismate via isochorismate^8^,^10^. The second pathway is also shared by some bacteria. For example, *Pseudomonas aeruginosa* produces SA via two distinct enzymes: isochorismate synthase (ICS) and isochorismate pyruvate lyase^11^. ICS homologues have been identified in a wide variety of plant species and genetic analyses revealed that the ICS-dependent SA biosynthetic pathway predominates under (a)biotic stresses in *Arabidopsis*, although *IPL* genes have not yet been identified in plants^8^,^10^.

Isochorismate is a precursor of both SA and many other distinct derivatives in plants, fungi and bacteria^10^. For example, in bacteria, isochorismate can be converted into 2,3-dihydro-2,3-dihydroxybenzoate (DDHB) and pyruvate by isochorismatase (ISC, EC 3.3.2.1). *Escherichia coli* EntB and *Vibrio cholerae* VibB contain a conserved amino-terminal ISC domain and carboxy-terminal aryl carrier protein domain. They play roles in the production of enterobactin and vibriobactin, respectively^12^,^13^. *Pseudomonas aeruginosa* PhzD is required for phenazine biosynthesis; it is 46% identical to EntB, but it lacks its C-terminal domains. PhzD also catalyses the hydrolysis of isochorismate^14^. Isochorismatase-like hydrolases have been identified from the pathogenic protozoans *Leishmania donovani, L. major* and *Trypanosoma cruzi*^15^, several phytopathogenic fungi^16^ and the rice root nematode *Hirschmanniella oryzae*^17^. However, their functions in these pathogens remain unknown.

Here we identified two genes from *P. sojae* (*PsIsc1*) and *V. dahliae* (*VdIsc1*) whose putative encoded proteins share high similarity with known ISCs and exhibit ISC activity. We demonstrated that both genes were required for virulence and that they function by suppressing SA accumulation in their hosts. The genes encode unconventionally secreted proteins and PsIsc1 could mediate the translocation of the C terminus of the *P. sojae* effector Avr1b into host cells. The *in planta* expression of these genes reduced SA levels. We propose that these two unconventionally secreted effectors hydrolyse host isochorismate, thereby modulating the SA metabolism pathway and suppressing host immunity.

## Results

### PsIsc1 and VdIsc1 are putative ISCs

As ISC can hydrolyse isochorismate (the direct precursor of SA), we hypothesized that some phytopathogens might exploit ISCs as virulence effectors to suppress host SA biosynthesis. Therefore, we searched for *P. sojae* and *V. dahliae* ISCs using reciprocal BLAST searches with 40 known ISCs from the PDB database (Supplementary Table 1). Of 17 candidates, three each from *P. sojae* and *V. dahliae* were identified as putative ISCs based on HHpred searches^18^ (Supplementary Table 2). Of these 6 candidate genes, one from each species (*PsIsc1* and *VdIsc1*) was upregulated during the infection stages (Supplementary Fig. 1). The predicted PsIsc1 and VdIsc1 proteins shared high primary and secondary structural similarities with the reported ISCs (Supplementary Fig. 2). Thus, they are candidate virulence proteins in these two filamentous pathogens.

### PsIsc1 is a virulence factor in *P. sojae*

**Figure 1:**
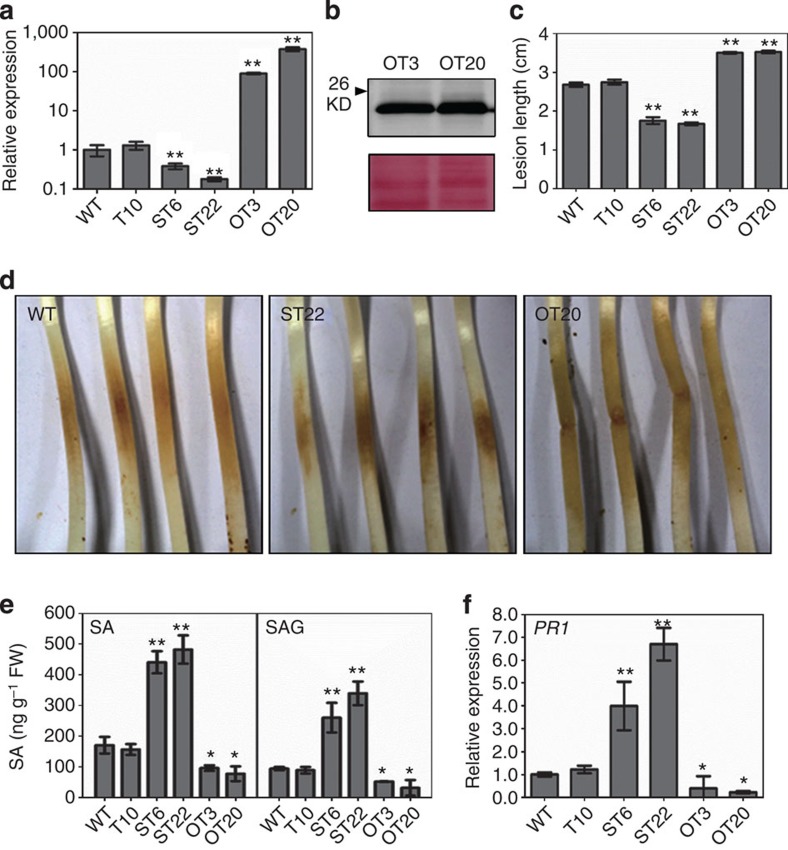
PsIsc1 is a virulence factor in *P. sojae*. (**a**) Relative levels of *PsIsc1* transcripts in *P. sojae* transformants measured using qRT–PCR. Relative transcript levels were standardized using *P. sojae actin* gene. T10 showed no significant transcriptional changes and served as a control. (**b**) Western blotting of proteins from *P. sojae* transformants expressing PsIsc1-His (OT3 and OT20). Anti-His antibody was used and a band at the expected size was detected (upper panel). Total proteins were stained using Ponceau S (lower panel). (**c**) Lesion lengths of soybean-etiolated seedling hypocotyls inoculated with 100 zoospores (36 hpi). (**d**) Phenotypes of soybean-etiolated seedlings inoculated with *P. sojae* transgenic lines. Typical photos taken at 36 hpi are shown. (**e**) Free SA and SAG levels (12 hpi) in soybean tissues infected with the WT strain and *P. sojae* transformants. FW, fresh weight. (**f**) Relative expression of soybean *PR1* in plants infected with the WT strain and transgenic lines at 12 hpi. Expression was normalized against soybean *actin*. In **a**–**f**, bars represent the s.e. from at least three independent replicates. Asterisks indicate significant differences (*P<0.05; **P<0.01; Dunnett’s test) between each transgenic line and the WT control.

To determine the role of *PsIsc1* in *P. sojae* virulence, we silenced or overexpressed the gene using polyethylene glycol-mediated protoplast transformation^19^. The *PsIsc1* transcripts were reduced to 38% and 18% in two silenced transgenic lines (ST6 and ST22, respectively) and increased by 90- and 376-fold in two overexpression transgenic lines (OT3 and OT20, respectively) (Fig. 1a). Western blotting confirmed that PsIsc1 was successfully expressed at the expected size in OT3 and OT20 (Fig. 1b). All transformants showed normal filamentous growth. Compared with the wild-type (WT) strain and a transgenic line (T10) with a normal *PsIsc1* expression level, the two silenced lines (ST6 and ST22) showed a visible reduction in virulence on soybean seedlings, whereas the two overexpressed lines (OT3 and OT20) were more virulent than the controls (Fig. 1c,d).

To further characterize PsIsc1-mediated virulence, we measured the SA levels in soybean seedlings infected with the *P. sojae* transformants. *PsIsc1* silencing increased the concentrations of both free SA and its glucoside (SAG) by ~3-fold. In contrast, *PsIsc1* overexpression significantly decreased the concentrations of SA and SAG (Fig. 1e). The transcript levels of the SA-inducible *PATHOGENESIS-RELATED 1* (*PR1*) gene were significantly increased or reduced accordingly at sites inoculated with the transformants compared with WT and T10 (Fig. 1f). Collectively, our results suggest that PsIsc1 functions as a virulence factor in *P. sojae* by suppressing host SA accumulation.

### VdIsc1 is required for full virulence in *V. dahliae*

**Figure 2:**
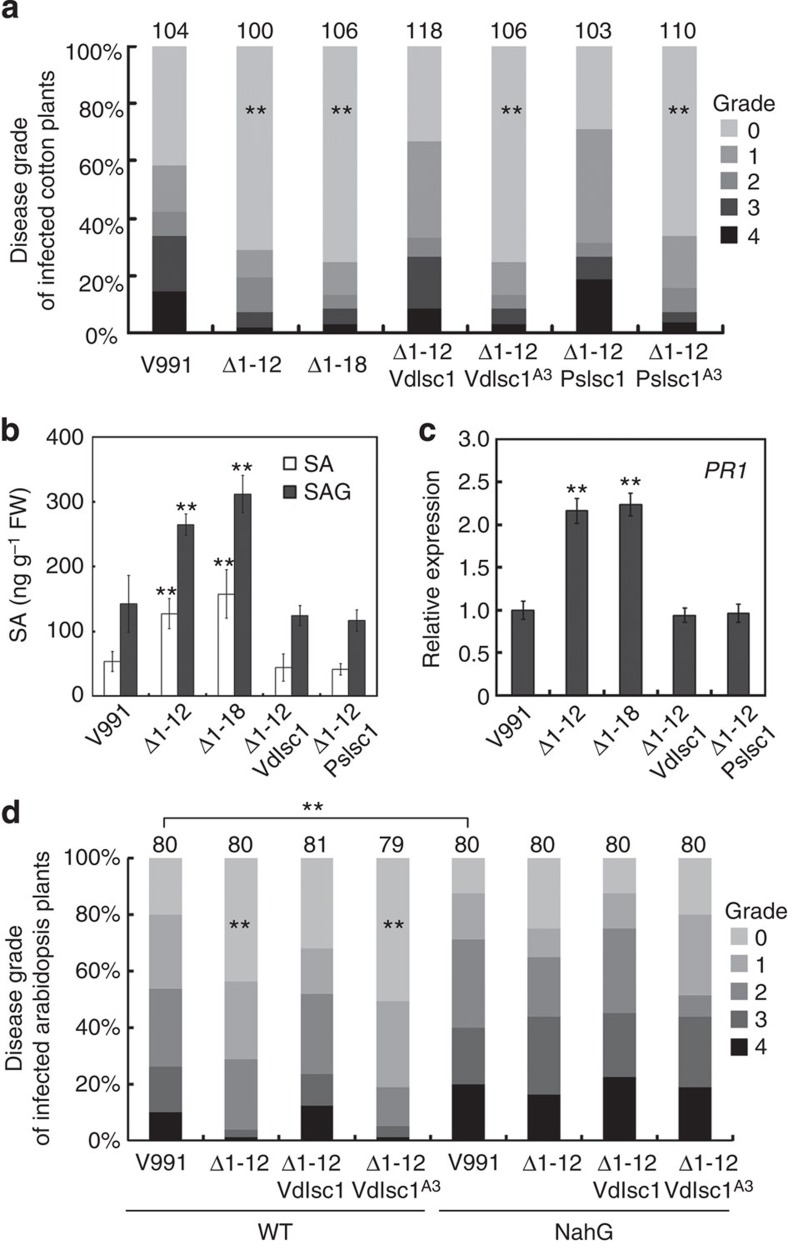
Functional VdIsc1 or PsIsc1 is required for full *V. dahliae* virulence. (**a**) Disease symptoms on cotton plants were scored at 15 dpi with the indicated strains. The disease grade is depicted in Supplementary Fig. 3. The total number of infected plants is indicated above each column (**P<0.01; *t*-test). V991 represents the WT *V. dahliae* isolate; Δ1-12 (V991ΔVdIsc1-12) and Δ1-18 (V991ΔVdIsc1-18) represent two strains in which *VdIsc1* was deleted; Δ1-12VdIsc1, Δ1-12VdIsc1^A3^, Δ1-12PsIsc1 and Δ1-12PsIsc1^A3^ represent, respectively, Δ1-12 complemented with *VdIsc1*, with a *VdIsc1* mutant encoding three key amino acid substitutions (D26A, K100A and C133A), with *PsIsc1* and with a *PsIsc1* mutant encoding comparable substitutions (D25A, K90A and C124A; Supplementary Fig. 2). All complementing constructs used the *Aspergillus nidulans trpC* promoter. (**b**) Free SA and SAG levels in cotton roots at 14 dpi. (**c**) Relative levels of cotton *PR1* transcripts measured using qRT–PCR at 12 hpi. Expression was normalized against the constitutively expressed cotton gene *Histone3*. In **b** and **c**, the data represent the mean±s.d.; **P<0.01 (Dunnett’s test) between WT and each transgenic line. (**d**) Disease symptoms of the *Arabidopsis* WT and *NahG*-transformed plants. The disease grades are described in the Methods. The total number of infected plants is shown above each column (**P<0.01; *t*-test).

To explore the role of *VdIsc1* in *V. dahliae* virulence, deletions were generated in *V. dahliae* strain V991 to produce two mutants: Δ1-12 (V991Δ*VdIsc1*-12) and Δ1-18 (V991Δ*VdIsc1*-18). Two mutants exhibited normal growth phenotypes (Supplementary Fig. 4). Based on pathogenicity tests in the cotton cultivar Lumian 21, the two *VdIsc1* deletion mutants produced significantly reduced disease symptoms. To confirm that the mutant phenotypes resulted from the deletion of *VdIsc1*, a single copy of the gene was introduced into Δ1-12. The resulting complemented strain, Δ1-12VdIsc1, was fully pathogenic and induced disease at rates similar to the WT strain. We also explored the ability of *PsIsc1* to complement the Δ1-12 deletion. *PsIsc1* could functionally replace *VdIsc1* (Fig. 2a and Supplementary Fig. 4), suggesting that the two genes play similar roles in virulence.

In cotton plants inoculated with the two deletion mutants, the free SA and SAG levels were increased by 100–150% (Fig. 2b) and there was a corresponding ~2-fold increase in the level of cotton *PR1* mRNA (Fig. 2c). The concentration of SA, SAG and *PR1* transcript levels after infection with the *VdIsc1-* and *PsIsc1-*complemented transformants were comparable with the WT (Fig. 2b,c). Thus, both genes can act as virulence factors in *V. dahliae* by reducing host SA levels. Both PsIsc1 and VdIsc1 contain a triad of catalytic residues (Asp-Lys-Cys; D-K-C), which form the activate sites and are responsible for the features of many known ISCs^15^,^20^ (Supplementary Fig. 2). We generated substitution mutations of the three key amino acids in both genes (*PsIsc1*^*A3*^ and *VdIsch1*^*A3*^) and found that neither mutant could complement the *V. dahliae* Δ*VdIsc1* mutant based on virulence tests (Fig. 2a), suggesting the virulence contributions of these effectors are probably linked to their enzymatic activities.

To elucidate the biological significance of the elevated SA levels during infection with the deletion mutants, we estimated the effect of SA on cotton resistance. The results revealed that pre-treatment with SA reduced virulence, comparable to Δ1-12 infections (Supplementary Fig. 5). This suggests that SA enhances the resistance of cotton toward *V. dahliae*. Furthermore, we analysed the virulence contributions of VdIsc1 using the *Arabidopsis thaliana* WT and *NahG* transformant plants. SA levels are extremely low in *Arabidopsis* plants that expresses the *Pseudomonas putida NahG* gene, because it encodes salicylate hydroxylase, an enzyme that converts SA to catechol^21^. As shown in Fig. 2d, the *NahG* transformant was significantly more susceptible to *V. dahliae* than *A. thaliana* WT, supporting the hypothesis that SA is required for plant resistance to *V. dahliae*. Δ1-12 or Δ1-12VdIsc^A3^, but not Δ1-12VdIsc1, exhibited significantly reduced virulence in *A. thaliana* WT. In contrast, the *NahG* transformant showed comparable susceptibility to *V. dahliae* WT, Δ1-12, Δ1-12VdIsc1 and Δ1-12VdIsc^A3^. Taken together, these results suggest that VdIsc1 suppress plant SA-mediated immunity, which could be a direct consequence of its inability to interfere with SA biosynthesis in the host plants.

### PsIsc1 and VdIsc1 are unconventionally secreted proteins

**Figure 3:**
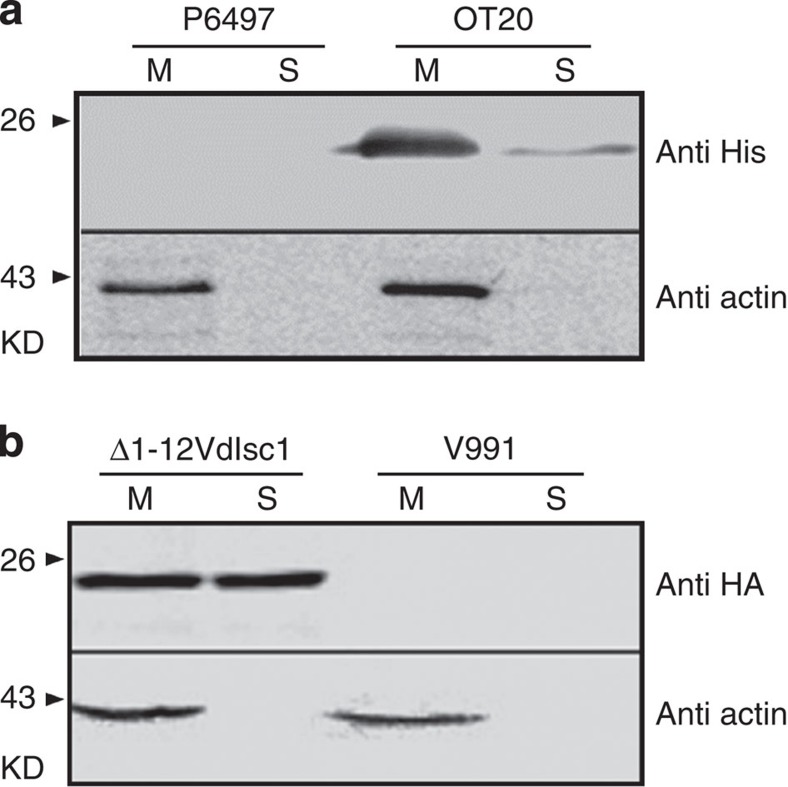
PsIsc1 and VdIsc1 are secreted proteins. PsIsc1-His (**a**) and VdIsc1-HA (**b**) were expressed in *P. sojae* (OT20) and *V. dahliae* (Δ1-12VdIsc1), respectively. Proteins extracted from mycelia (M) and culture supernatants (S) were analysed using western blotting with anti-His, -HA or -α-actin antibodies, as indicated. Extracts and supernatants from non-transformed *P. sojae* P6497 and *V. dahliae* V991 were used as controls for antibody specificity.

To identify the site of action of VdIsc1 and PsIsc1 during interactions, we analysed whether they were secreted. Neither protein was predicted to contain an N-terminal signal peptide that could direct the protein to the conventional secretory pathway (Supplementary Table 2 and Supplementary Fig. 2). However, both were predicted to be non-classically secreted based on SecretomeP 2.0 (ref. 22), with NN scores (neural network output scores) of 0.66 and 0.81, respectively (Supplementary Table 2), which is above the threshold of 0.5. To test the predictions experimentally, we used western blotting to detect secreted proteins in culture supernatants of the *P. sojae* transgenic line OT20 and the *V. dahliae* complemented line Δ1-12VdIsc1, which produced His-tagged PsIsc1 and haemagglutinin (HA)-tagged VdIsc1, respectively. PsIsc1-His and VdIsc1-HA could be detected in culture supernatants, as well as in hyphae of the respective transformants. However, the control cytoplasmic protein, α-actin, was only found in the hyphae of both cases (Fig. 3). Thus, PsIsc1 and VdIsc1, although they lack classical N-terminal signal peptides, are secreted proteins.

### PsIsc1 is translocated into host cells during interactions

**Figure 4:**
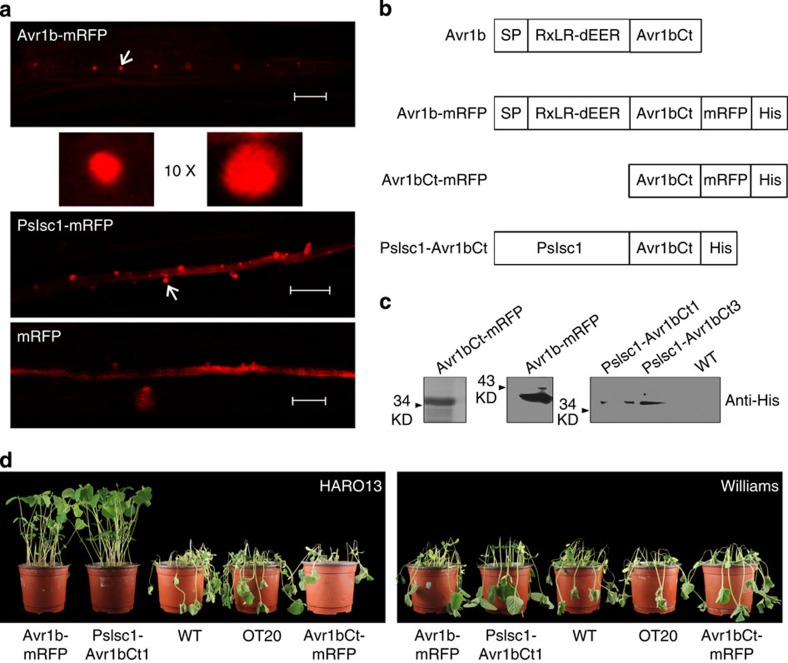
PsIsc1 can replace the translocation domain of Avr1b. (**a**) PsIsc1 preferentially accumulates in haustoria. *P. sojae*-infected hyphae were observed using confocal microscopy at 10 hpi. *P. sojae* expressing Avr1b-mRFP showed specific haustorial accumulation (indicated by the arrow) of fluorescent Avr1b (red, upper panel), whereas PsIsc1-mRFP accumulated preferentially in haustoria (red, middle panel). P6497 expressing mRFP served as a control (lower panel). Scale bars, 20 μm. The arrow indicated haustoria were shown with tenfold magnification. (**b**) Structure of the fusion of full-length PsIsc1 and the C terminus of Avr1b (Avr1bCt: aa, 66–138, removing the signal peptide and RxLR-dEER domain). (**c**) Western blotting of proteins from *P. sojae* transformants expressing Avr1b-mRFP, Avr1bCt-mRFP and PsIsc1-Avr1bCt (PsIsc1-Avr1bCt1 and PsIsc1-Avr1bCt3). The *P. sojae* isolate P6497 served as a control for antibody specificity. (**d**) The phenotypes of hypocotyls from soybean cultivars HARO13 (*Rps*1b) and Williams (*rps*). Photographs were taken 2 dpi.

To assess the localization of PsIsc1 during infection, we generated the *P. sojae* transformants producing the coding region of monomeric red fluorescent protein (mRFP) fused to the C terminus of PsIsc1 or the *P. sojae* RxLR effector Avr1b^19^. PsIsc1-mRFP fusion protein was validated in both mycelia and culture supernatants, and was the same as the Avr1b-mRFP. In contrast, soluble protein green fluorescent protein (GFP) was detected in mycelia, but not in the culture supernatants (Supplementary Fig. 6). These results further indicate that PsIsc1 and Avr1b are secreted proteins. Avr1b-mRFP was predominantly localized in haustoria and the extrahaustorial matrix during soybean infection. Fluorescence was not detected within infected cells, presumably due to the dilution of Avr1b-mRFP within the plant cells (Fig. 4a), as observed with *P. infestans* Avr3a^23^. PsIsc1-mRFP also showed strong localization in haustoria and the extrahaustorial matrix, but was not detected in plant cells. The unfused control (mRFP) did not show haustorial localization (Fig. 4a). Therefore, PsIsc1 is distributed in a similar manner as the host cytoplasmic effector Avr1b, and both proteins might be secreted from haustoria during interactions between *P. sojae* and soybean.

To determine whether PsIsc1 enters host cells following secretion, we examined whether it could deliver Avr1b into host soybean cells. Oomycete RxLR effectors such as the *P. infestans* effector Avr3a^23^ and *P. sojae* Avr1b^19^,^24^ depend on their N-terminal RxLR-dEER domain for host cell delivery. We demonstrated previously that the transformants expressing WT Avr1b lost the ability to infect soybean plants carrying *Rps*1b but retained the ability to infect plants lacking *Rps*1b, and that the N-terminal RxLR-dEER domain was required its translocation into host cells^19^. We deleted the N-terminal region from Avr1b (producing Avr1bCt) and replaced it with PsIsc1 (producing PsIsc1-Avr1bCt), and then generated two *P. sojae* transformants that expressed the fused PsIsc1-Avr1bCt (Fig. 4b). Production of the expected proteins was confirmed using western blotting (Fig. 4c). Transformants expressing the fused Avr1b-mRFP were consistently avirulent on soybean cultivars carrying *Rps*1b (Fig. 4d), suggesting that the recognition of Avr1b inside soybean cells was not affected by the fusion with mRFP. The inoculation of soybean hypocotyls showed that the two transformants expressing PsIsc1-Avr1bCt were unable to infect the soybean cultivar HARO13 containing the *Rps*1b resistance gene, but could still infect Williams, which lacks *Rps*1b. In contrast, the recipient WT strain (P6497) and the transgenic lines expressing PsIsc1 (OT20) or Avr1bCt-mRFP could kill both cultivars (Fig. 4d). Similar results were observed using a leaf inoculation assay (Supplementary Fig. 7). Taken together, these data suggest that PsIsc1 could functionally replace the secretory leader and host cell entry domain of Avr1b, and that PsIsc1 might be translocated inside host cells during infection.

### PsIsc1 and VdIsc1 suppress SA-mediated defense *in planta*

**Figure 5:**
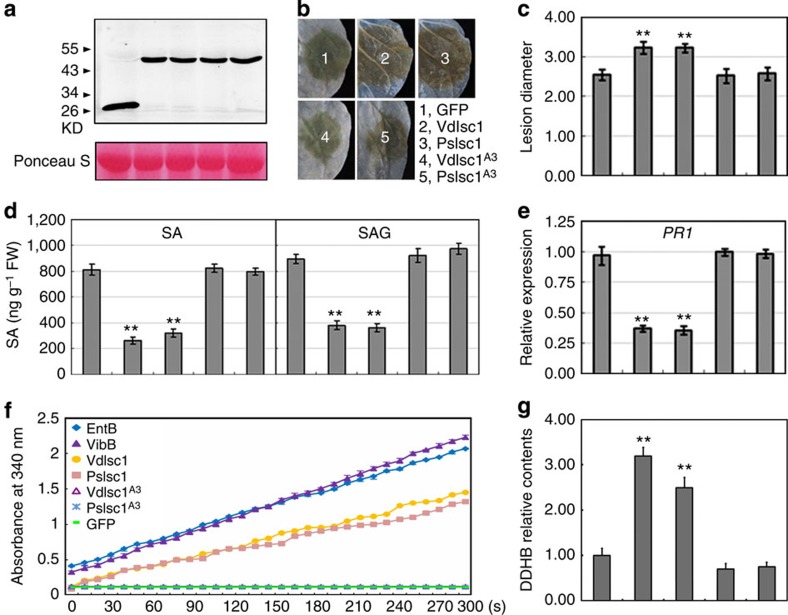
PsIsc1 and VdIsc1 suppress disease resistance. (**a**) Western blotting of the indicated transiently expressed proteins in *N. benthamiana* using anti-GFP antibodies. (**b**) Phenotypes of leaves transiently expressing the indicated genes inoculated with *P. capsici* zoospores. Photos were taken at 48 hpi. (**c**) Lesion diameters of infected regions at 48 hpi, averaged from at least 30 inoculated sites. (**d**) Free SA (left) and SAG (right) levels (12 hpi) in leaves transiently expressing the indicated genes. (**e**) Relative levels of *PR1* gene transcripts at 12 hpi in infected leaves transiently expressing the indicated genes. (**f**) Isochorismatase activity by measuring the absorbance at 340 nm. Bacterial EntB and VibB produced by *E. coli* were used as positive controls. An identical quantity of the tested proteins was purified from plant tissues. VibA generates NADH from DDHB and NAD^+^ when active ISC was present (producing DDHB from isochorismate); the absorbance of NADH at A340 nm at the indicated time points (seconds) was recorded. (**g**) Relative DDHB concentrations in plant tissues expressing the indicated genes. The relative DDHB concentration of each tested sample was calculated based on A340 absorbance and the DDHB concentration in GFP-expressing leaves was normalized to 1.0. In **a**, **c**–**e** and **g**, from left to right: GFP, VdIsc1, PsIsc1, VdIsc1^A3^ and PsIsc1^A3^. **P<0.01 (Dunnett’s test).

To determine whether PsIsch1 and VdIsch1 function within plant cells to compromise immunity, we expressed these two genes (as *GFP* fusions) transiently in *Nicotiana benthamiana* tissue using *Agrobacterium* infiltration. Western blotting with anti-GFP antibodies confirmed the expression of proteins of the expected length (Fig. 5a). We also assessed the distribution of PsIsc1 and VdIsc1 in plant cells. Neither of the two proteins were co-localized with the mCherry-tagged plastid marker pt-rk CD3-999 (ref. 25), but were instead localized to the cytoplasm and nucleus of transformed *N. benthamiana* cells, similar to native GFP alone (Supplementary Fig. 6). We then inoculated leaves with a compatible pathogen, *P. capsici*. Compared with leaves expressing *GFP* alone, the diameters of the lesions caused by *P. capsici* were significantly increased following expression of *PsIsc1* and *VdIsc1* (Fig. 5b,c). The levels of SA (Fig. 5d) and *PR1* mRNA (Fig. 5e) in the inoculated sites were reduced when the two genes were expressed, indicating that PsIsc1 and VdIsc1 suppressed SA-mediated *Phytophthora* resistance in *N. benthamiana*. When mutations were introduced into the catalytic triads of PsIsc1 and VdIsc1, they no longer suppressed resistance to *P. capsici* or reduced SA and *PR1* transcript levels when transiently expressed in *N. benthamiana* (Fig. 5).

### PsIsc1 and VdIsc1 have ISC activity

To examine whether PsIsc1 and VdIsc1 could catalyse hydrolysis of isochorismate into DDHB, we purified targeted proteins from *N. benthamiana* leaves (Fig. 5a) and used *E. coli* EntB and *V. cholerae* VibB that were expressed in *E. coli* as positive controls. The enzyme activity was determined as described previously by measuring the increase in absorbance at 340 nm caused by NAD^+^ reduction^13^. *In planta*-expressed PsIsc1 and VdIsc1, as well as two positive controls, exhibited high enzyme activity. Mutations in the catalytic triads of PsIsc1 and VdIsc1 resulted in an almost complete loss of enzyme activity. No activity was detected when GFP (a negative control) was purified and analysed using the same procedures (Fig. 5f). We also measured the relative levels of DDHB in plant tissues expressing the above genes. Significantly increased DDHB concentrations were detected in total extracts of the leaves expressing *PsIsc1* or *VdIsc1*, whereas DDHB accumulated at relatively lower levels in leaves expressing *GFP*, *PsIsc1*^*A3*^ or *VdIsch1*^*A3*^ (Fig. 5g). Therefore, both proteins have ISC activity.

### N termini of PsIsc1 and VdIsc1 are required in *V. dahilae*

**Figure 6:**
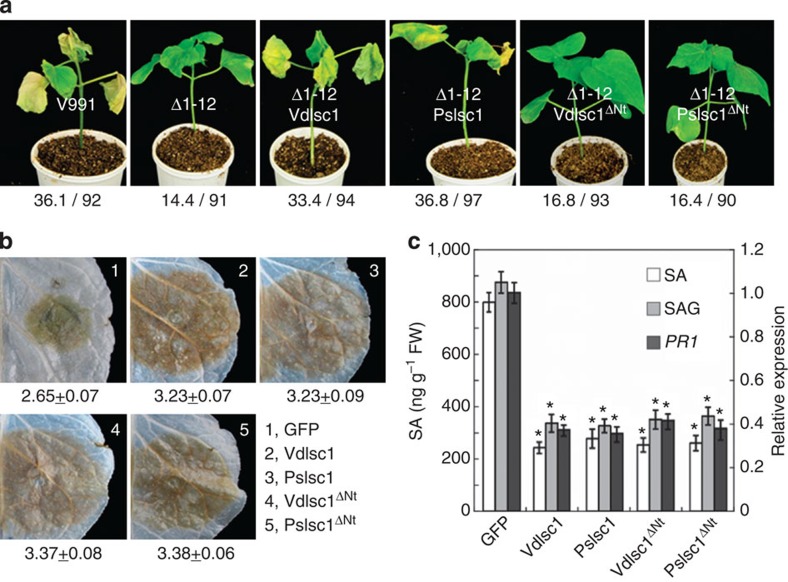
Functional analysis of the N termini of PsIsc1 and VdIsc1. (**a**) Phenotypes of cotton seedlings inoculated with the indicated strains. The photos were taken at 15 dpi and a typical seedling is shown as an example for each line. The numerator indicates the average disease index, which is calculated based on the disease grades of the individual inoculated seedlings and the total number (denominator). (**b**) The disease symptoms of leaves transiently expressing the indicated genes that were inoculated with *P. capsici* zoospores. Photos were taken at 48 hpi. The numbers indicate the lesion diameters of infected regions that were scored at 48 hpi and represent the mean at least 30 inoculated sites. (**c**) The relative levels of SA, SAG (left axis) and *PR1* transcripts (right axis) at 12 hpi in infected leaves transiently expressing the indicated genes. *P<0.01 (Dunnett’s test).

PsIsc1 and VdIsch1 contain no motifs that are similar to RxLR^19^,^23^. We constructed mutants of both proteins lacking the N-terminal regions (PsIsch1Δ^Nt^ and VdIsch1Δ^Nt^) (Supplementary Fig. 2). In these mutants, the NN scores calculated using the SecretomeP 2.0 server were below the normal threshold for secreted proteins^22^ (Supplementary Fig. 2). Neither of the deletion mutants complemented the phenotypes of *VdIsc1*-deletion mutants in cotton seedlings (Fig. 6a), suggesting that both proteins require their putative unconventional secretion signals to function in *V. dahliae*. To confirm that loss of the N-terminal regions did not compromise ISC activity, we transiently expressed *PsIsc1*Δ^Nt^ and *VdIsc1*Δ^Nt^ in *N. benthamiana* leaves. The deletion mutants conferred similar phenotypes as their WT in terms of the levels of resistance (Fig. 6b), the SA/SAG content and *PR1* transcripts (Fig. 6c). Therefore, the N-terminal region is probably required for the secretion of these proteins from pathogens, but not for their roles in plant cells.

## Discussion

Little is known about how filamentous plant pathogens interfere with host innate immunity for successful colonization and infection. As SA-mediated signalling pathways are important for plant immune responses and isochorismate is a precursor of SA, it has been inferred that phytopathogens could produce ISCs to reduce SA accumulation in response to pathogen attack and thus inhibit plant defense responses^16^,^17^,^26^. In the current study, we have demonstrated experimentally that *P. sojae* and *V. dahliae* encode ISC effectors (PsIsc1 and VdIsc1) to disrupt the SA metabolism pathway. Notably, we provide evidence that the ISCs are probably secreted from the two filamentous pathogens through unconventional pathways, and that PsIsc1 can enter into host cells during infection.

It was reported that ISC family motifs were found in the secretome of some phytopathogenic filamentous ascomycete fungi, but not in non-phytopathogenic species^16^. Isochorismatase hydrolase was also identified specifically in a highly aggressive isolate of *V. dahliae* using proteomics-based analyses^26^. We identified putative ISC genes in *P. sojae* and *V. dahliae* (*PsIsc1* and *VdIsc1*, respectively) based on primary and secondary structural similarities with known ISCs and demonstrated that both genes were highly expressed during infection stages. The overexpression or silencing of *PsIsc1* in *P. sojae* led to increased and reduced virulence, respectively; SA levels in infected host tissues were altered accordingly. Similar results were observed when *VdIsc1* was deleted in *V. dahliae*. Interestingly, the *PsIsc1* gene could functionally replace *VdIsc1* in *V. dahliae*. The transient overexpression of these two genes in *N. benthamiana* tissues suppressed SA levels and SA-mediated defense; consistent with this, DDHB levels were increased. Furthermore, we demonstrated that the conserved ISC catalytic triads in PsIsc1 and VdIsc1 were required for ISC activity and the suppression of plant immunity. Thus, we conclude that these two evolutionary distinct pathogens both generate ISCs to promote infection by destroying a precursor (isochorismate) of the plant defense-signalling hormone SA.

**Figure 7:**
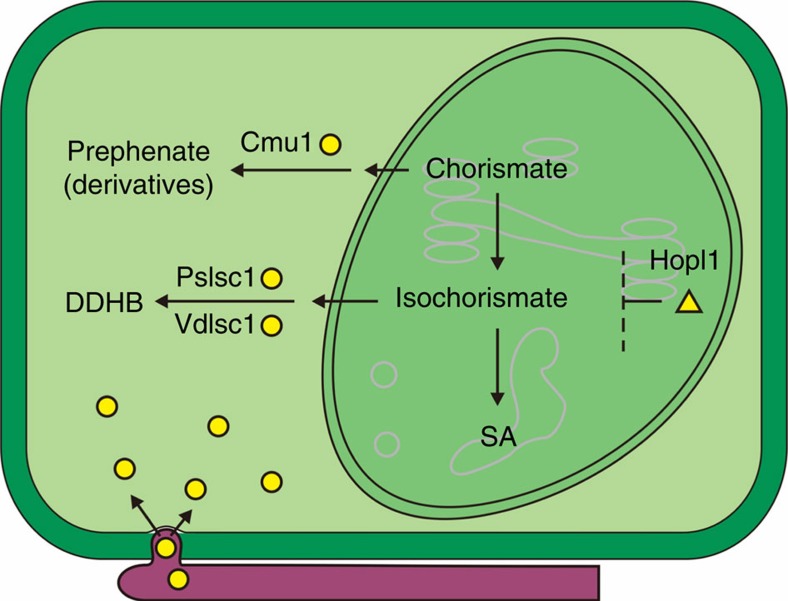
Pathogen effectors subverting plant SA biosynthesis. Pathogens use cell-entering effectors (yellow) to suppress plant SA biosynthesis in distinct manners. Filamentous pathogen-derived enzymatic effectors (circle) inhibit SA accumulation by redirecting its precursors from plastid (darker green) to cytosol (mint green), whereas bacterial effector HopI1 (triangle) depresses SA levels by remodelling chloroplast thylakoid structure.

As summarized in Fig. 7, several distinct mechanisms have been adopted by phytopathogens to suppress host SA biosynthesis by cell-entering effectors. The *Pseudomonas syringae* effector HopI1 remodels host chloroplast thylakoid structure and suppresses SA accumulation via an unknown mechanism^27^. *Ustilago maydis* secretes a host cytoplasmic effector (Cmu1), which is a chorismate mutase that converts chorismate to prephenate. As chorismate is a key intermediate in the biosynthesis of SA^10^, *U. maydis* can effectively deplete chorismate and suppress SA accumulation via Cmu1 (ref. 28). Secreted chorismate mutases are found in many plant fungal and nematode pathogens^28^,^29^. In the current study, we demonstrated that phytopathogens from two kingdoms have evolved a novel strategy to inhibit SA biosynthesis in plants by producing ISC effectors. It is likely to be that neither of the pathogen-generated ISCs are imported into the plastids of host plants, because neither PsISC1 nor VdISC1 were co-localized with the plastid marker pt-rk CD3-999 (ref. 25) when they were transiently co-expressed in the leaves of *N. benthamiana* (Supplementary Fig. 8). However, when delivered into host plant cells by the pathogens, the ISC effector proteins might be able to affect the cellular homeostasis of isochorismate. Although SA is thought to be synthesized in the plastids, plastid-produced isochorismate may transport to the cytosol where it acts as the substrate of VdIsc1 or PsIsc1 to be catalysed into DDHB. Reduction of cytosolic concentrations of isochorismate by pathogen-derived ISC effectors in turn may enhance its export from the plastid to the cytosol. A similar mechanism was also proposed to explain how *U. maydis* CMU1 acts on plant chorismate^28^.

Secreted proteins containing ISC family motifs have been found in several fungal and nematode pathogens^16^,^17^, suggesting a common strategy for host manipulation. Therefore, different phytopathogens have evolved distinct intracellular effectors independently to actively suppress SA accumulation in host cells. The findings suggest a promising strategy for disease control by inhibiting the activities of these enzymatic effectors or supplying the host with plant-defense activators to compensate for the SA shortage caused by pathogen effectors. For example, several highly active inhibitors targeting the secreted chorismate mutase from *Mycobacterium tuberculosis* have been designed and evaluated based on structure-guided approaches^30^. Our results suggest that secreted ISCs from phytopathogens could be suitable targets for disease control.

We further showed that PsIsc1 and VdIsc1 are secreted proteins, although neither protein contained a predicted signal peptide when we analysed using two widely used algorithms^31^,^32^. Proteins that lack signal peptides can access the cell exterior via the unconventional protein secretion pathway, which has been documented in animal, plant, bacterial and yeast cells^33^,^34^. Indeed, both proteins were predicted to be unconventionally secreted proteins using an available algorithm^22^. The secretion mode of ISCs suggests that unconventional secretion occurs in *P. sojae* and *V. dahliae*. At least four distinct transport pathways are involved in unconventional secretion^34^, but it remains unknown which (if any) of these mechanisms are used by these two filamentous pathogens. The recruitment of unconventional secretion for effector delivery in both an oomycete and a fungal plant pathogen further broadens the extensive resources pathogens can bring to bear against hosts, and broadens the range of proteins that bioinformaticians must examine as potential effectors.

We used *P. sojae* to examine the pathway of PsIsc1 secretion and host entry in more detail. We demonstrated that PsIsc1 accumulates predominantly in haustoria and the extrahaustorial matrix during soybean infection, which is a similar distribution to that of well-characterized cell-entering effectors, such as *P. sojae* Avr1b^19^ and *P. infestans* Avr3a^23^. We did not observe the translocation of fluorescent protein fusions of PsIsc1 inside host cells during infection, which might be due to dilution of the fluorescence signal in plant cell cytoplasm. Nevertheless, we demonstrated that PsIsc1 could functionally replace the N-terminal regions (host target signals, including the signal peptide and RxLR-dEER domain) in Avr1b and mediate the translocation of Avr1b into host cells, consistent with the prediction that PsIsc1 enters into host cells during infection to metabolize the SA precursor. The observation that PsIsc1 was functional when expressed in *V. dahliae* suggests that either PsIsc1 does not rely on any pathogen-encoded machinery for entry, or that the two pathogens produce functionally analogous translocation machinery.

Assessing how the effectors from filamentous pathogens are translocated into host cells is a major focus of current research. In oomycete pathogen effectors, the RxLR-dEER domain is considered to mediate host cell entry by binding to phosphatidylinositol phosphates on the outer surface of plant plasma membranes^24^. Consistent with this, *Phytophthora* pathogens could generate external phosphatidylinositol-3-phosphate to promote the entry of RxLR effectors^35^. In contrast, *Magnaporthe oryzae* intracellular effectors accumulate preferentially in the biotrophic interfacial complex, a novel interfacial structure that is associated with the initial invading hyphae^36^,^37^, suggesting that the effectors may be translocated through a specialized structure in this fungal pathogen. In the current study, we showed that both PsIsc1 and VdIsc1 probably function inside host cells and PsIsc1 accumulates in haustoria. Nevertheless, the exact mechanism behind the translocation is yet to be investigated.

In summary, these results provide evidence that *P. sojae* and *V. dahliae* produce a novel class of effectors: ISC enzymes that destroy the precursor of the plant defense-signalling hormone SA. The discovery of these enzymes in both fungi and oomycetes suggests that this is a central mechanism of virulence in filamentous plant pathogens. We also demonstrated that unconventional secretion has been recruited by plant pathogens in two kingdoms to deliver effectors into host plants; in addition to the biological novelty, this discovery transforms the way that candidate effectors should be identified, which is at present heavily reliant on the identification of signal peptides.

## Methods

### Bioinformatic analyses

Known ISC protein sequences were obtained from the PDB database ( http://www.rcsb.org)^38^ and were used as queries to search against the *P. sojae*^1^ and *V. dahliae*^2^ protein sequence databases using reciprocal Blast searches programmes. All the obtained proteins were analysed using HHpred^18^ against the PDB and PFAM databases ( http://pfam.sanger.ac.uk/)^39^. Proteins were considered to be putative ISCs only if they met the following criteria: in a search of the PFAM database, the proteins were classified as a member of the PF00857 family, which groups together ISCs; and the HHpred searches identified the proteins as belonging to the ISC family (the top ten hits contain over five ISCs). The putative ISCs identified were further confirmed using annotations in the NCBI database.

Sequence alignments were generated using the MUSCLE algorithm^40^. Secondary structure was predicted using Porter ( http://distill.ucd.ie/porter/)^41^ and then compared with the known crystal structures of ISCs from *Oleispira antarctica* (PDB code: 3LQY)^20^, *Leishmania* major (PDB code: 1XN4) and *Trypanosoma cruzi* (PDB code: 1YZV)^15^. Signal peptide prediction was performed using SignalP 4.1 ( http://www.cbs.dtu.dk/services/SignalP/)^32^ and iPSORT ( http://ipsort.hgc.jp/)^31^. Non-classically secreted proteins were predicted using SecretomeP 2.0 ( http://www.cbs.dtu.dk/services/SecretomeP/) with the threshold of neural network output score of 0.5 as recommended by the algorithm^22^.

### Microbial strains and plant lines

All of the pathogen strains, the derived transgenic *P. sojae* and *V. dahliae* lines, and plant materials used in the current study are summarized in Supplementary Table S3. *P. sojae* and *P. capsici* strains were grown and maintained as described previously^35^. *V. dahliae* strain V991, a highly toxic defoliating WT strain, was grown on PDA medium at 25 °C^42^. *E. coli* and *Agrobacterium tumefaciens* were grown using appropriate selectable markers. All plants were grown in growth chambers at appropriate temperatures with 16-h photoperiods and 55% humidity.

### Generation of transformants and virulence assays

Transformed *P. sojae* lines were obtained using polyethylene glycol-mediated protoplast transformation procedure^19^,^35^. Putative transformants were screened by quantitative reverse transcriptase (qRT)–PCR to identify silenced or overexpression transgenic lines. The overexpression lines were confirmed by western blotting using antibodies against the relevant tags. The pathogenicity phenotypes of selected *P. sojae* transformants were determined by hypocotyl inoculation of etiolated soybean seedlings. Approximately 100 zoospores of each transformant and the WT strain were inoculated onto the hypocotyl of etiolated (susceptible Williams) soybean seedlings. The avirulence phenotypes of selected transformants were evaluated using hypocotyl inoculation^19^ with the soybean cultivars Williams (*rps*) and HARO13 (Harosoy background, *Rps*1b). Each avirulence or virulence determination was repeated at least three times. Significant differences were identified using Dunnett’s test.

*V. dahliae* transformation was performed using *Agrobacterium tumefaciens*-mediated transformation^43^. Briefly, the conidia (1 × 10^8^ spores per ml) were harvested and then mixed with an equal volume of *A. tumefaciens* containing the appropriate vector and 200 μM acetosyringone. After incubation, the cultures were transferred to PDA medium containing 50 μg l^−1^ hygromycin B (Roche, Switzerland). The putative transformants were screened for gene deletions or the presence of transgenes using genomic PCR and qRT–PCR. For complementation, the mutant strains were further transformed with *VdIsc1*, *PsIsc1* and the different mutants fused with an HA tag. For cotton and *Arabidopsis* infection assays, plants were inoculated with *V. dahliae* WT or transformants by conidial root dip^42^. For *Arabidopsis* inoculations, we modified the dip time to 5 min and the inoculum to 1 × 10^7^ conidia per ml. The symptoms were evaluated and the disease grade was classified as follows: 0 (no symptoms), 1 (>0–25% wilted leaves), 2 (25–50%), 3 (50–75%) and 4 (75–100%). The data were analysed using Student’s *t*-test.

### Molecular experiments

Promoters, flanking sequences, complete open reading frames and/or fragments of *PsIsc1*, *VdIsc1*, *Avr1b* and *EntC* were amplified by PCR using specific primers (Supplementary Table 4) and cloned via standard restriction digest and ligation methods to produce expression constructs based on the vectors pC1300, pTOR, pET21b, pBinGFP2 or pBinGFP4 (Supplementary Table 5).

Total RNA was extracted from cotton roots using the CTAB method^42^. Total RNA was extracted from *N. benthamiana* leaves, etiolated soybean seedlings, and *P. sojae* and *V. dahliae* mycelium using RNA simple Total RNA Kit (Tiangen, China) following the manufacturer’s instructions. qRT–PCR was performed using SYBR green real-time RT-PCR assays (TaKaRa) as described previously^35^. To assess the transcriptional profile of genes from *P. sojae*, total RNA was collected from *P. sojae* P6497 mycelium sandwiched between pairs of soybean leaves. The mycelium was harvested at 2, 4 and 10 h after inoculation (hpi) and the infected soybean leaves, including mycelia, were collected at 12, 24, 36 and 48 hpi. To assess the transcriptional profile of genes from *V. dahliae* inoculum, mycelia were inserted into wounded *N. benthamiana* petioles and the mycelia were recovered at 6, 12, 24 hpi, and the infected petioles, including mycelia, were also collected at 3 and 5 dpi.

### Western blotting

Protein extracts were prepared by grinding ~100 mg leaf or slightly dried mycelia samples in liquid nitrogen. The resulting powder was then suspended in protein extraction buffer (50 mM HEPES, 150 mM KCL, 1 mM EDTA, 0.1% Triton X-100, pH 7.5) supplemented with 1 mM dithiothreitol and 1 × protease inhibitor cocktail (Roche). The resultant suspensions were mixed and centrifuged at 12,000*g* at 4 °C for 15 min and the supernatants were recovered. Samples were fractionated using standard SDS–PAGE gels (12% acrylamide) and blotted using standard procedures^44^. Anti-His-tag primary monoclonal antibody (Sigma-Aldrich) and IRDye 800CW-conjugated goat (polyclonal) anti-mouse IgG (H+L; LI-COR Biosciences) secondary antibodies were used to assess *P. sojae* transformants. Anti-GFP monoclonal antibody (Sigma-Aldrich) and IRDye 800CW-conjugated goat anti-mouse IgG secondary antibodies were used to assess transient gene expression in plants. All the primary antibodies were diluted with 5% (m/V) non-fat dried milk in PBS (1:3,000) and the secondary antibodies were diluted with 5% (m/V) non-fat dried milk in PBS (1:10,000). The membranes were then visualized using a LI-COR Odyssey scanner with excitation at 700 and 800 nm. The molecular marker is shown in Supplementary Fig. 6 and blots were shown with at least one size-labelled marker.

*V. dahliae* strain Δ1-12VdIsc1, *P. sojae* strains OT20, PsIsc1-mRFP, and Avr1b-mRFP and GFP were used to analyse the secretion of VdIsc1 and PsIsc1. The proteins were purified from the same amounts of culture (including the mycelia and supernatants) for each strain. For *P. sojae*, mycelia were cultivated in flasks at 25 °C with shaking at 100 r.p.m. for 2 weeks in liquid Czapek culture medium. Culture supernatants were precipitated using ammonium sulphate and then analysed using western blotting with mouse anti-His (Sigma-Aldrich) and anti-α-actin antibodies (Sigma, A3853). For *V. dahliae*, mycelia were cultivated in a flask for 2 weeks at 25 °C in liquid Czapek–Dox medium and the secreted proteins were collected following the reported procedures^45^. Culture supernatants were precipitated using trichloroacetic acid and then analysed using western blotting with mouse-anti HA (Sigma, H3663) and anti-α-actin antibodies.

### *N. benthamiana* infiltration and virulence assays

*A. tumefaciens* strain GV3101 containing the appropriate vector was infiltrated into *N. benthamiana* leaves^35^. *P. capsici* infection assays were performed using droplet inoculations of zoospore solutions of *P. capsici* isolate 35 (10 μl, ~500 zoospores) onto detached *N. benthamiana* leaves. At least ten independent (4-week-old) *N. benthamiana* leaves were tested per construct combination. To explore the effect of transient expression on infection, proteins were first expressed by infiltrating cells of the relevant *A. tumefaciens* strain into *N. benthamiana* leaves 24 h before *P. capsici* inoculation. The growth efficiency of *P. capsici* was quantified by measuring the lesion diameter (mm) at 48 hpi.

### Measurement of SA/SAG concentrations and application

SA was extracted from leaves excised from soybean, cotton and *N. benthamiana* plants, and quantified using HPLC as described^46^ with minor modifications. Briefly, leaf powder (~1.0 g) was ground in liquid nitrogen and then suspended in 90% (v/v) methanol. As an internal standard for SA, 100 μg 3-hydroxy benzoic acid in 100% methanol were added to each sample. The SA solution was filtered and separated on a C18 analytical column using HPLC and detected using fluorescence (excitation at 305 nm, emission at 405 nm; Waters). The HPLC was programmed for isocratic conditions with a flow rate of 0.5 ml min^−1^. To assay SAG, the aqueous phase containing SAG was acidified to pH 1.0 using HCl and was then boiled for 30 min to release SA from any acid-labile conjugated forms. The released SA was extracted using an organic mixture and treated as described above. SA and SAG were quantified by area integration of the HPLC peaks.

Cotton plants were sprayed with 2.0 mM SA 1 day before inoculation or were mock treated with water before infection. Following inoculation, the plants were sprayed with SA every 7 dpi.

### Enzyme activity assays and measurement of DDHB content

Isochorismatase activity was measured as described previously^13^. Briefly, the enzyme EntC was used to convert chorismate to isochorismate and enzyme activity was then determined by measuring the increase in absorbance at 340 nm caused by the production of NADH from NAD^+^ when *V. cholerae* VibA was present in the reaction solution (converting DDHB+NAD^+^ to 2,3-DHB+NADH) using an Uvikon spectrophotometer. Purified *E. coli* EntB and *V. cholerae* VibB produced from *E. coli* were used as positive controls^13^. The tested proteins were purified from *N. benthamiana* transiently expressing *VdIsc1* or *PsIsc1* genes using HisTrap HP (GE Healthcare) following the manufacturer’s instructions. The assay was performed in a total volume of 100 μl containing 10-μl reaction solutions from the first step, 100 mM PBS buffer pH 7.0, 0.8 mM NAD^+^, 4 μg of each purified protein and 3.8 μg of VibA at room temperature.

To measure the relative DDHB concentration in plant tissues, the crude extracts were collected from ~1 g *N. benthamiana* leaves. The assay was performed in a total volume of 100 μl containing 100 mM PBS, pH 7.0, 10-μl crude extracts, 3.8 μg purified VibA, 0.8 mM NAD^+^, 100 mM PBS buffer pH 7.0 at room temperature for 10 min. Next, the absorbance at 340 nm owing to NAD^+^ reduction was used to calculate the relative DDHB contents in the tested samples.

### Microscopy

One hundred zoospores from each *P. sojae* transformant were inoculated onto the hypocotyl of the susceptible soybean Williams etiolated seedlings. The surface layer of the inoculated tissue was then examined ~10 hpi. GFP-VdIsc1 and GFP-PsIsc1 fusions were expressed together with the mCherry-labelled plastid localization marker (pt-rk CD3-999)^25^ in *N. benthamiana* leaves. Protein fluorescence in the *N. benthamiana* leaves was observed using a ZEISS LSM 710 confocal microscope (Zeiss Microsystems) with a × 40 objective lens (Zeiss) at the specific excitation and emission wavelengths (RFP, 561 and 600–650 nm; GFP, 488 and 495–530 nm; mCherry, 587 and 600–650 nm).

## Author contributions

T.L., T.S., H.B., L.S., W.L., J.X., S.L., L.C., T.C. and M.Z. performed the wet bench experiments. All authors contributed to experiment design and data analysis. D.D., T.L., T.S. and X.Z. wrote the manuscript with input from all authors. D.D. directed the project.

## Additional information

**How to cite this article:** Liu, T. *et al.* Unconventionally secreted effectors of two filamentous pathogens target plant salicylate biosynthesis. *Nat. Commun.* 5:4686 doi: 10.1038/ncomms5686 (2014).

## Supplementary Material

Supplementary Information Supplementary Figures 1-8 and Supplementary Tables 1-5
