# Supplementary material for: Unconventionally secreted effectors of two filamentous pathogens target plant salicylate biosynthesis
Source: Nat Commun. 2014 Aug 26;5:4686. doi: 10.1038/ncomms5686 (PMC4348438; doi:10.1038/ncomms5686)
Supplement: Supplementary Information — Supplementary Figures 1-8 and Supplementary Tables 1-5 [file ncomms5686-s1.pdf]

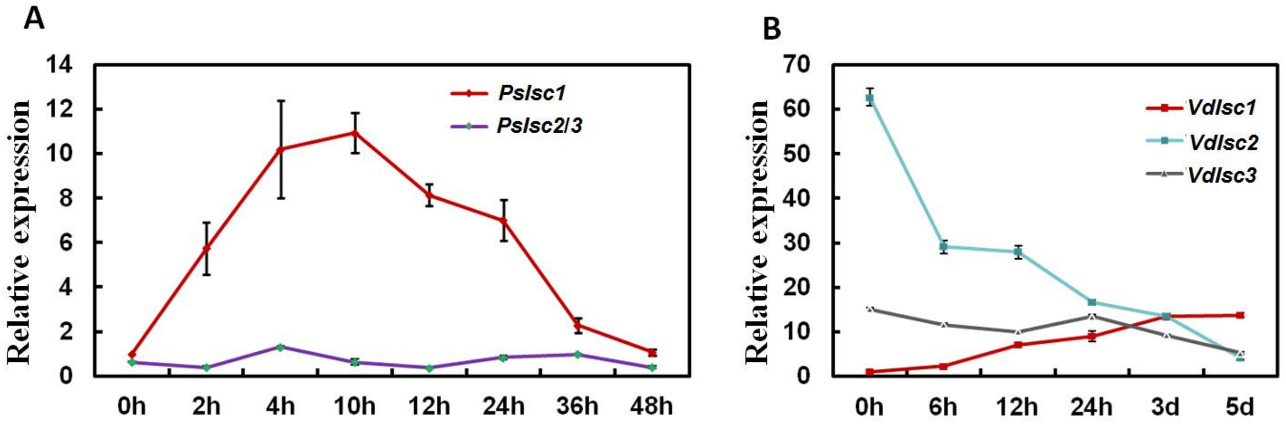

Supplementary Figure 1: *PsIsc1* and *VdIsc1* are up-regulated during infection

The transcriptional profiles of three predicted isochorismatase genes in *P. sojae* (**A**) and *V. dahliae* (**B**). The relative levels of each transcript were measured using qRT-PCR at the indicated time points post-infection. The *P. sojae* and *V. dahliae actin* genes were used as references. Susceptible soybean cultivar Williams was used for *P. sojae* infection, and *N. benthamiana* petioles were used for *V. dahliae*.

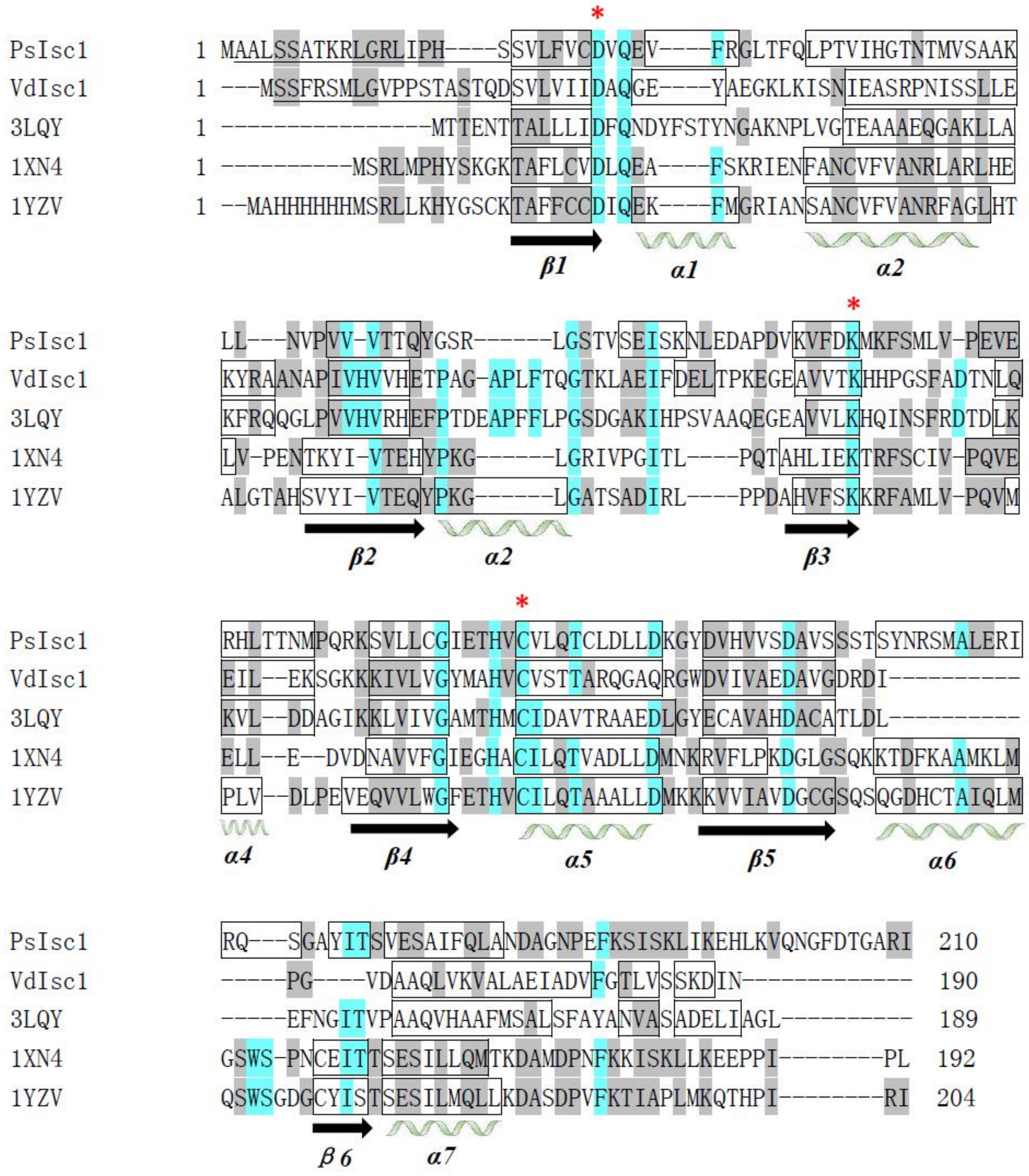

Supplementary Figure 2: Bioinformatic analysis of PsIsc1 and VdIsc1

The amino acid sequences of the PsIsc1 and VdIsc1 were aligned with those of known isochorismatases from *Oleispira antarctica* (PDB code: 3LQY), *Leishmania* major (PDB code: 1XN4), and *Trypanosoma cruzi* (PDB code: 1YZV) using MUSCLE. PsIsc1 and VdIsc1 display 29.7% and 33.5% sequence identity to the well-characterized *O. antarctica* isochorismatase, respectively. The conserved residues are highlighted in cyan (highly conserved) and gray (moderately conserved). The predicted secondary structures of PsIsc1 and VdIsc1, predicted using the Porter algorithm, are boxed; they share high similarity with the well-characterized crystal structure of known isochorismatases. Predicted ß-strands (ß) and α-helices (α) are indicated. The conserved catalytic triad of residues essential for isochorismatases that were mutated in the current study is marked with red asterisks. The N-terminal regions deleted in this study are underlined.

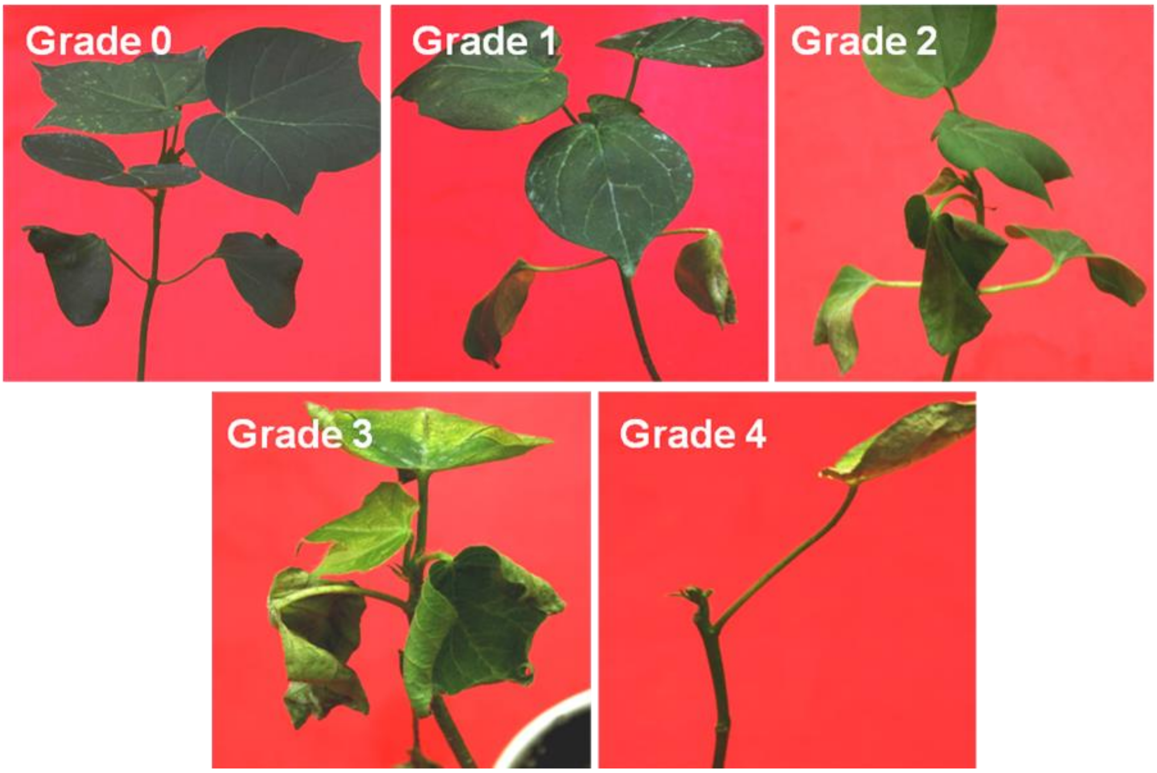

Supplementary Figure 3: Examples of cotton *Verticillium* wilt disease ratings

Grade 0, healthy plant, no symptoms; Grade 1, only one or two wilted or detached cotyledons; Grade 2, only one true leaf showing wilting symptoms or detached; Grade 3, two or more true leaves showing wilting symptoms or detached, but some healthy leaves remaining; Grade 4, no healthy leaves remaining, or the plant was dead.

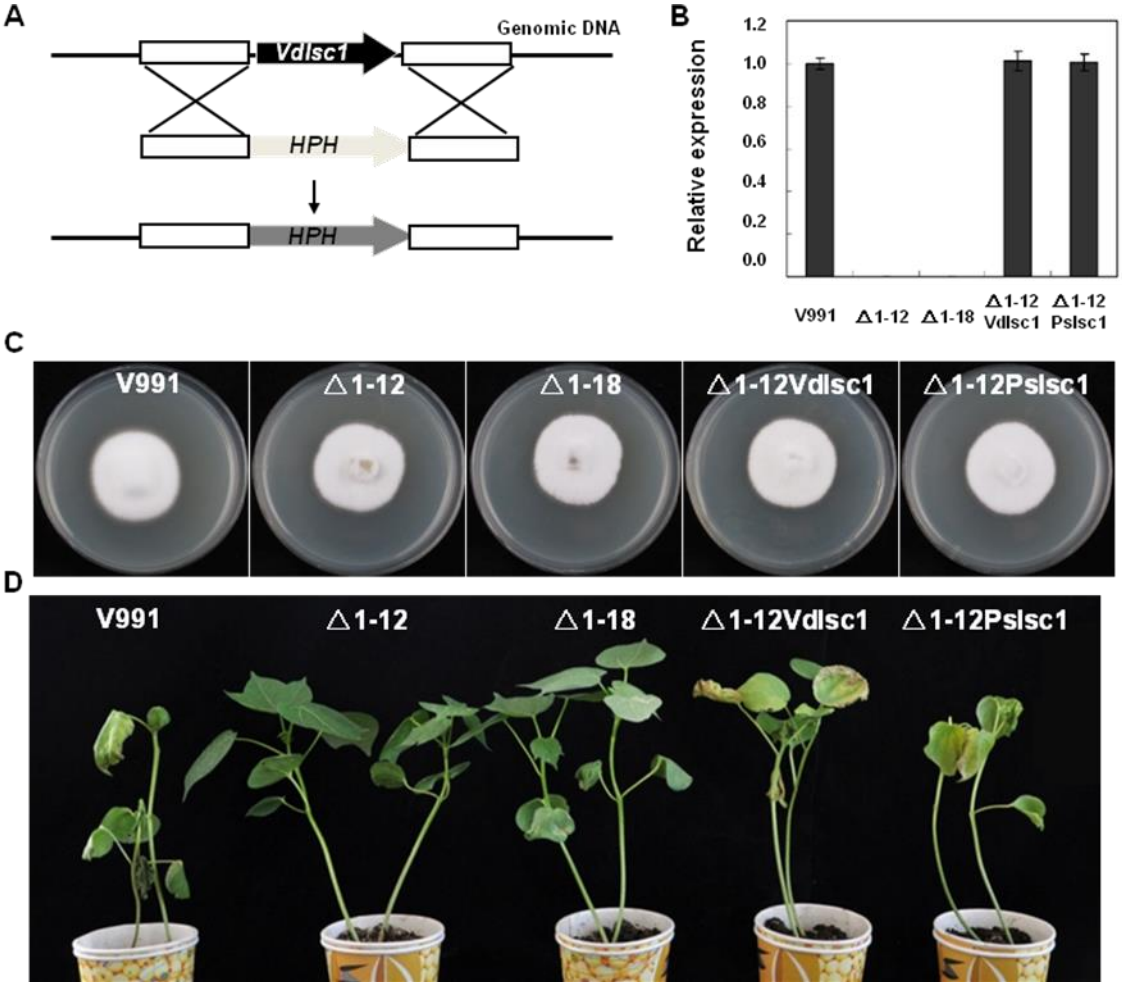

Supplementary Figure 4: Generation of *V. dahliae* transformants and virulence assays

(**A**) *VdIsc1* targeted gene replacement strategy. A 630-bp fragment of the VdIsc1 coding region was replaced with a 2.1-kb fragment containing the hygromycin B-resistance cassette (HPH) to create the VdIsc1 mutants. (**B**) Confirmation of gene knockout and complementation using qRT-PCR analysis. The relative levels of *VdIsc1* were measured using qRT-PCR. The *V. dahliae actin* gene was used as reference. (**C**) Colony morphology of *V. dahliae* transformants observed after 6 days at 25ºC in the dark. (**D**) Examples of disease symptoms of cotton seedlings infected with the indicated *V. dahliae* strains. Photos were taken at 15 dpi.

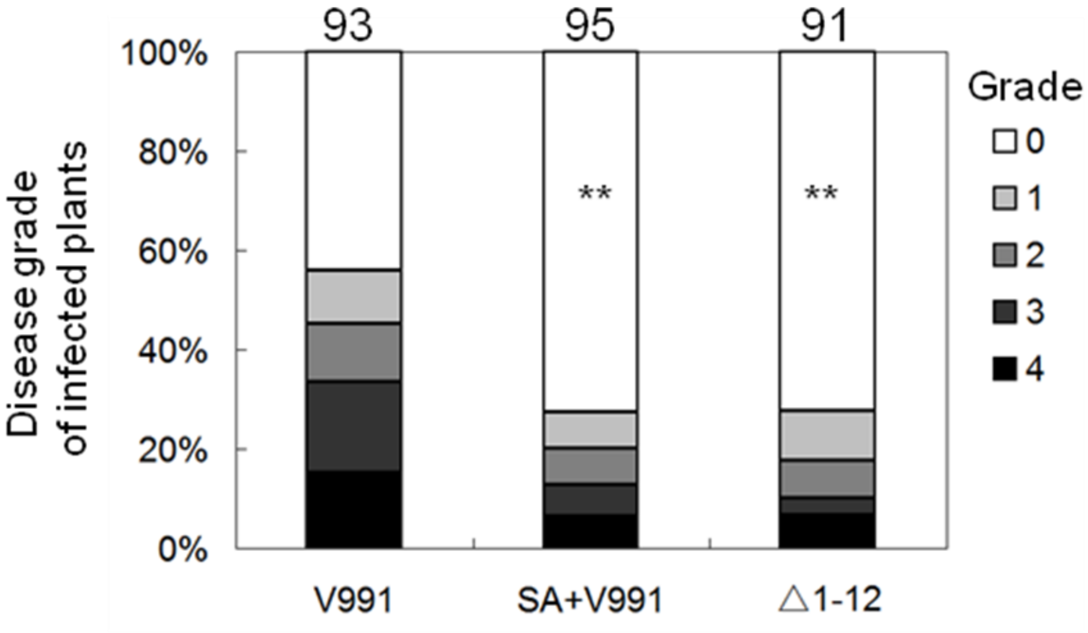

Supplementary Figure 5: Effect of SA application on the resistance of cotton

Cotton plants were sprayed with 2.0 mM SA 1 day prior to inoculation with *V. dahliae* V991, or were mock treated with water prior to infection. Following inoculation, the plants were sprayed with SA every 7 dpi. Disease symptoms were scored at 15 dpi using the methods described in Fig. 2A.

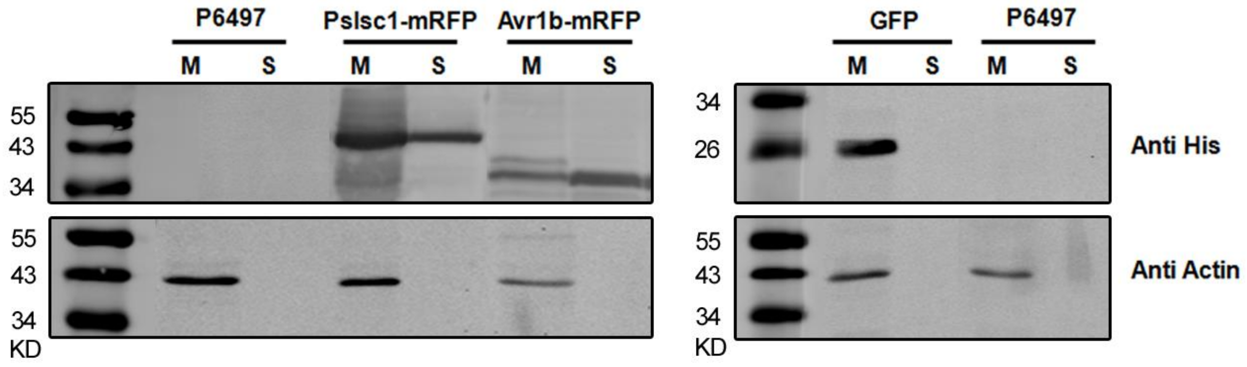

Supplementary Figure 6: PsIsc1 is a secreted protein

Western blotting of PsIsc1, Avr1b, and GFP from mycelia (M) and culture supernatants (S). PsIsc1-mRFP, Avr1b-mRFP, and GFP were expressed in *P. sojae*. Proteins extracted from mycelia and culture supernatants were analyzed using Western blotting with anti-His, or -α-actin antibodies, as indicated. Extracts and supernatants from non-transformed *P. sojae* P6497 were used as controls for antibody specificity.

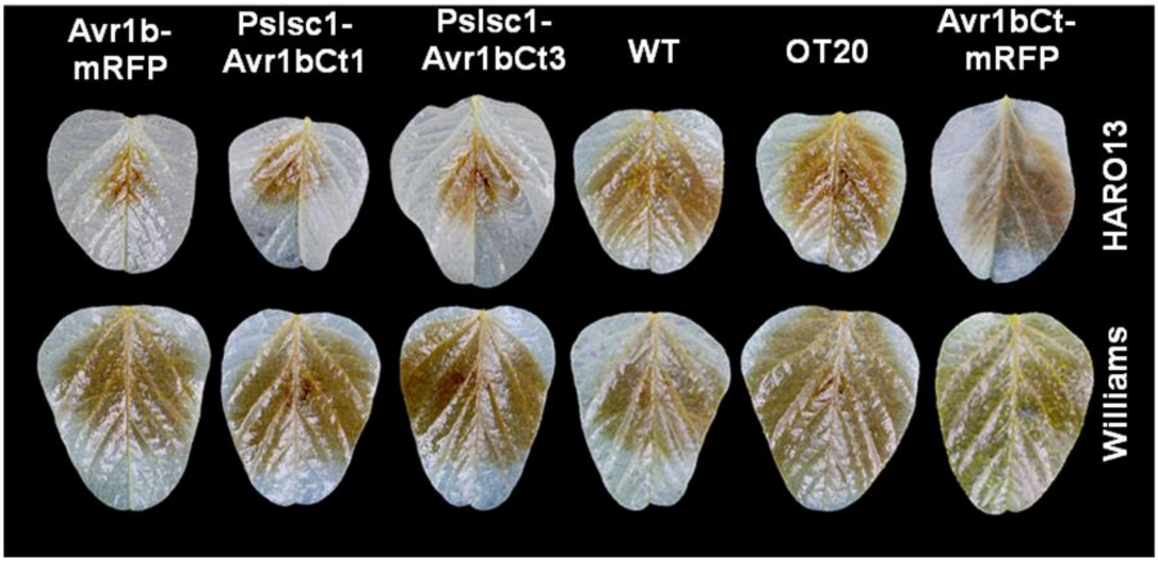

Supplementary Figure 7: Phenotypes of inoculated soybean leaves

Soybean cultivars HARO13 (*Rps*1b) and Williams (non *rps*) were used and photographs were captured at 2 dpi from the indicated strains.

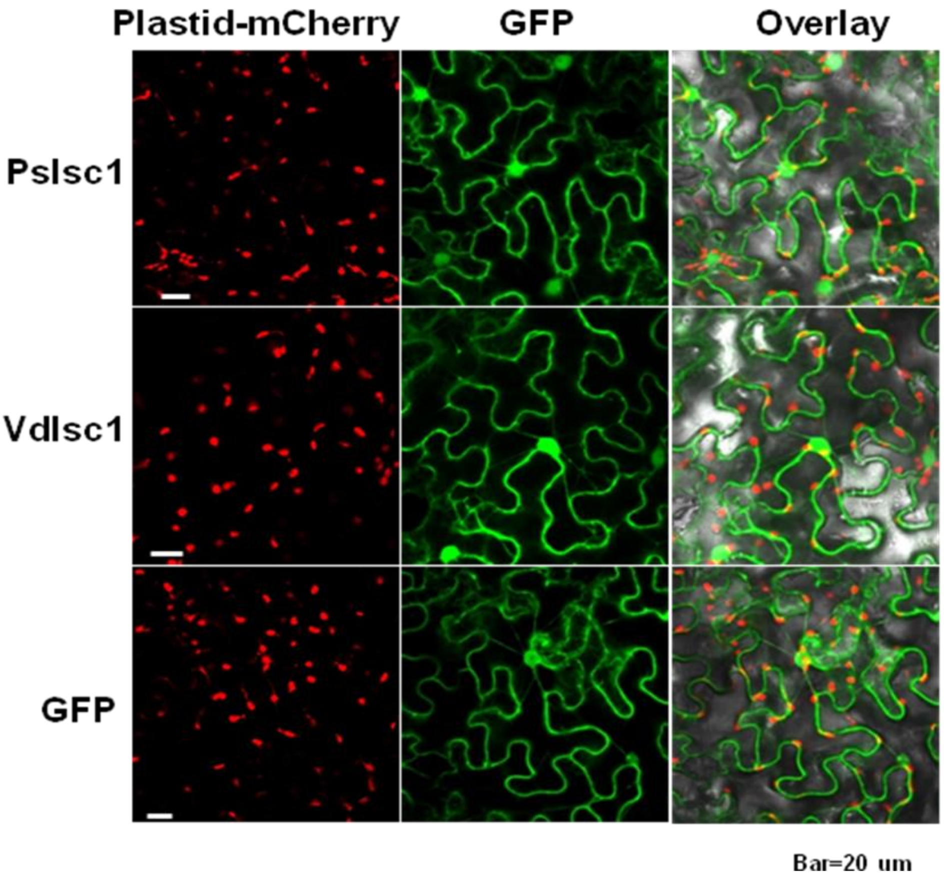

Supplementary Figure 8: Nuclear and cytoplasmic localization of PsIsc1 and VdIsc1

N. benthamiana leaves expressing *GFP* fused to the indicated genes together with the mCherry-labeled plastid localization marker pt-rk CD3-999 were observed using laser scanning microscopy. Bar=20 um.

Supplementary Table 1: Lists of query sequences used in this study

PDB ID [1]	DB ID [2]	Organism	Chain Length	Protein Name
1ILW	O58727	*Pyrococcus horikoshii*	180	
1IM5	O58727	*Pyrococcus horikoshii*	180	
1NF8	Q7DC80	*Pseudomonas aeruginosa*	207	PhzD
1NF9	Q7DC80	*Pseudomonas aeruginosa*	207	
1X9G	D0VWV0	*Leishmania donovani*	200	
1XN4	Q4QGT7	*Leishmania major*	192	
1YZV	Q4D3U8	*Trypanosoma cruzi*	204	
2WTA	B0VA03	*Acinetobacter baumannii*	235	
3IRV	Q48J46	*Pseudomonas syringae*	233	
3LQY	3LQY	*Oleispira antarctica*	189	OaIHL
3PL1	Q50575	*Mycobacterium tuberculosis*	186	
3TB4	P0C6D3	*Vibrio cholerae*	223	
3TG2	P0C6D3	*Vibrio cholerae*	223	
3TXY	Q2T332	*Burkholderia thailandensis*	195	
1YAC	P21367	*Escherichia coli*	208	
2FQ1	P0ADI4	*Escherichia coli*	285	EntB
2WT9	B0VA03	*Acinetobacter baumannii*	235	
3EEF	Q9HKY9	*Thermoplasma acidophilum*	182	
3HU5	Q72G28	*Desulfovibrio vulgaris*	204	
3MCW	Q7NYF4	*Chromobacterium violaceum*	197	
3OQP	Q13UL1	*Burkholderia xenovorans*	210	
3R77	Q51790	*Pseudomonas fluorescens*	207	
4H17	Q88LV1	*Pseudomonas putida*	196	
1J2R	P0ADI7	*Escherichia coli*	199	
1NBA	P32400	*Arthrobacter sp.*	264	
2A67	Q82ZG7	*Enterococcus faecalis*	166	
3O90	Q97PM2	*Streptococcus pneumoniae*	211	
3O91	Q97PM2	*Streptococcus pneumoniae*	211	
3O92	Q97PM2	*Streptococcus pneumoniae*	211	
3O93	Q97PM2	*Streptococcus pneumoniae*	211	
3O94	Q97PM2	*Streptococcus pneumoniae*	211	
3R2J	A4HRG8	*Leishmania infantum*	227	
3S2S	Q8DSG2	*Streptococcus mutans*	217	
2H0R	P53184	*Saccharomyces cerevisiae*	216	
3V8E	P53184	*Saccharomyces cerevisiae*	216	
2B34	Q20062	*Caenorhabditis briggsae*	199	
3HB7	A6TWT6	*Alkaliphilus metalliredigens*	200	
3OT4	Q7TTE5	*Bordetella bronchiseptica*	205	
3UAO	Q7TTE5	*Bordetella bronchiseptica*	205	
3KL2	Q82NB5	*Streptomyces avermitilis*	226	

Notes:

1. PDB ID from http://www.rcsb.org was obtained by searching for isochorismatase family (PF00857).
2. DB ID is a code for each entry in the UniProt sequence database (http://www.uniprot.org/)

Supplementary Table 2: Bioinformatic analysis of PsIsc1 and VdIsc1

Name[1]	General information[2]	Reciprocal Blast	Pfam[3]	Hhpred[4]	Annotation	Secretion[5]
PsIsc1	Protein ID: Ps108159	Query ID: 2B34	Family: ISC	ISC-hits: 6	SMART: ISC	SignalP: N
	Protein length: 210	E-value: 2.0E-37	Score: 100;<br>P-value: 1.6E-42	ISCF-hits: 4	E-value: 6.2E-32	Ipsort: N
	Genome location:<br>S1:1318273-1317537	Identity (%): 42	Identity (%): 31	OTH-hits: 0	NCBI: ISC (7.0E-137)	SecretomeP: Y
PsIsc2	Protein ID: Ps132163	Query ID: 3KL2	Family: ISC	ISC-hits: 7	SMART: ISC	SignalP: N
	Protein length: 340	E-value: 4.0E-08	Score: 100;<br>P-value: 4.6E-42	ISCF-hits: 3	E-value: 6.2E-16	Ipsort: N
	Genome location:<br>S19:571936-570840	Identity (%): 34	Identity (%): 28	OTH-hits: 0	NCBI: ISC (5.0E-26)	SecretomeP: N
PsIsc3	Protein ID: Ps144924	Query ID: 3KL2	Family: ISC	ISC-hits: 6	SMART: ISC	SignalP: N
	Protein length: 542	E-value: 6.0E-08	Score: 100;<br>P-value: 2.5E-39	ISCF-hits: 4	E-value:1.8E-15	Ipsort: Y
	Genome location:<br>S335:13845-12066	Identity (%): 34	Identity (%): 29	OTH-hits: 0	NCBI: ISC (6.0E-25)	SecretomeP: Y
Ps158850	Protein ID: Ps158850	Query ID: 3V8E	Family: ISC	ISC-hits: 1	SMART: ISC	SignalP: N
	Protein length: 615	E-value: 6.0E-08	Score: 100;<br>P-value: 6.0E-38	ISCF-hits: 9	E-value: 0.024	Ipsort: N
	Genome location:<br>S160:66456-63715	Identity (%): 27	Identity (%): 34	OTH-hits: 0	NCBI:<br>Nicotinamidase (1.0E-67)	SecretomeP: Y
Ps140706	Protein ID: Ps140706	Query ID: Ps140672	Family: ISC	ISC-hits: 1	SMART: d1jjya_	SignalP: Y
	Protein length: 249	E-value: 9.0E-87	Score: 99.9;<br>P-value: 3.6E-30	ISCF-hits: 9	E-value: 0.012	Ipsort:Y
	Genome location:<br>S88:283095-283994	Identity (%): 60	Identity (%): 21	OTH-hits: 0	NCBI:<br>Nicotinamidase (6.0E-46)	SecretomeP: Y
Ps140715	Protein ID: Ps140715	Query ID: Ps140672	Family: ISC	ISC-hits: 1	SMART: ISC	SignalP: Y
	Protein length: 316	E-value: 8.0E-137	Score: 100;<br>P-value: 1.8E-39	ISCF-hits: 9	E-value: 0.008	Ipsort: Y
	Genome location:<br>S88:306940-305842	Identity (%): 77	Identity (%): 26	OTH-hits: 0	NCBI:<br>Nicotinamidase (2.0E-64)	SecretomeP: N
Ps132531	Protein ID: Ps132531	Query ID: Ps140672	Family: LRR	ISC-hits: 0	SMART: Efh	SignalP: N
	Protein length: 1283	E-value: 3.0E-68	Score: 98.2;<br>P-value: 3.8E-14	ISCF-hits:0	E-value: 7.0E-06	Ipsort: N
	Genome location:<br>S21:394108-397959	Identity (%):31	Identity (%): 23	OTH-hits: 10	NCBI:<br>LRR (4.0E-31)	SecretomeP: N
Ps142274	Protein ID:Ps142274	Query ID:Ps140672	Family: LRR	ISC-hits:0	SMART:LRR	SignalP: N
	Protein length:1259	E-value:2.0E-153	Score:98.8;<br>P-value:3.0E-14	ISCF-hits:0	E-value:1.2E-5	Ipsort: N
	Genome location:<br>S111:41296-37425	Identity (%):31	Identity (%):23	OTH-hits:10	NCBI:<br>LRR(3.0E-59)	SecretomeP: Y

Ps14067 2	Protein ID:Ps140672	Query ID: 2H0R	Family: ISC	ISC-hits: 0	SMART: ISC	SignalP: N
	Protein length: 1628	E-value: 2.0E-06	Score: 100; P-value:1.8E-39	ISCF-hits:0	E-value: 0.005	Ipsort: N
	Genome location: S88:125598-131300	Identity (%):25	Identity (%):17	OTH-hits: 10	NCBI: Nicotinamidase(1.0E-59)	SecretomeP: N
VdIsc1	Protein ID: VDAG_05103	Query ID: 4H17	Family: ISC	ISC-hits:8	SMART: ISC	SignalP: N
	Protein length: 190	E-value: 8.0E-25	Score: 100; P-value:7.0E-47	ISCF-hits: 2	E-value: 4.4E-46	Ipsort: N
	Genome location: S1.9:431965-432593	Identity (%): 35	Identity (%):35	OTH-hits: 0	NCBI: ISC (3.0E-135)	SecretomeP: Y
VdIsc2	Protein ID: VDAG_03530	Query ID:3MCW	Family: ISC	ISC-hits:8	SMART: ISC	SignalP: N
	Protein length:230	E-value: 1.0E-22	Score: 100; P-value:3.4E-42	ISCF-hits:2	E-value: 2.8E-20	Ipsort: N
	Genome location: S1.5:1714583-1715516	Identity (%):35	Identity (%):33	OTH-hits: 0	NCBI: ISC (2.0E-166)	SecretomeP: Y
VdIsc3	Protein ID:VDAG_06346	Query ID:2B34	Family: ISC	ISC-hits:5	SMART: ISC	SignalP: N
	Protein length:203	E-value:3.0E-25	Score: 100; P-value:1.1E-42	ISCF-hits:5	E-value:2.2E-31	Ipsort: N
	Genome location: S1.13:699107-699898	Identity (%):36	Identity (%):31	OTH-hits: 0	NCBI: ISC (3.0E-146)	SecretomeP: Y
VDAG_ 06170	Protein ID:VDAG_06170	Query ID: 3V8E	Family: ISC	ISC-hits:2	SMART: ISC	SignalP: N
	Protein length:224	E-value:2.0E-35	Score: 100; P-value:1.1E-42	ISCF-hits:8	E-value:1.7E-23	Ipsort: N
	Genome location: S1.24:26094-26989	Identity (%):37	Identity (%):40	OTH-hits:0	NCBI: Nicotinamidase(5.0E-163)	SecretomeP: Y
VDAG_ 06688	Protein ID:VDAG_06688	Query ID:3HU5	Family: ISC	ISC-hits:4	SMART: ISC	SignalP: N
	Protein length:907	E-value:2.0E-06	Score: 100; P-value:3.0E-38	ISCF-hits:6	E-value:1.8E-20	Ipsort: N
	Genome location: S1.12:174463-175165	Identity (%):30	Identity (%):27	OTH-hits:0	NCBI: ISC (0)	SecretomeP:N
VDAG_ 08870	Protein ID:VDAG_08870	Query ID:1YAC	Family: ISC	ISC-hits:4	SMART: ISC	SignalP:Y
	Protein length:195	E-value:5.0E-10	Score:99.9; P-value:1.0E-28	ISCF-hits:6	E-value:1.2E-06	Ipsort:Y
	Genome location: S1.14:286966-290252	Identity (%):31	Identity (%):25	OTH-hits:0	NCBI: YcaC (6.0E-138)	SecretomeP: Y
VDAG_ 06924	Protein ID:VDAG_06924	Query ID: VDAG_05359	Family:Amidase	ISC-hits:0	SMART:Amidase	SignalP: N
	Protein length:1816	E-value:4.0E-59	Score:100; P-value:1.2E-84	ISCF-hits:0	E-value:4.4E-91	Ipsort: N
	Genome location: S1.15:117132-111621	Identity (%):37	Identity (%):35	OTH-hits:10	NCBI: Amidolyase (0)	SecretomeP: N

VDAG_ 05359	Protein ID:VDAG_05359	Query ID:3HU5	Family:Amidase	ISC-hits:0	SMART: ISC	SignalP: N
	Protein length:720	E-value:1.0E-8	Score:100; P-value:8.6E-90	ISCF-hits:0	E-value:3.3E-38	Ipsort: N
	Genome location: S1.10: 208699-210906	Identity (%):33	Identity (%):33	OTH-hits:10	NCBI: Amidolyase (0)	SecretomeP: Y

Notes:

1. Gene and protein names used in this study. Six isochorismatase encoding genes were identified based on the following analysis.
2. Protein ID and genome localization information were obtained from DOE Joint Genome Institute (http://genome.jgi.doe.gov) and Broad Institute (http://www.broadinstitute.org).
3. HHpred searching in Pfam protein family database (PfamA_27.0, http://pfam.sanger.ac.uk/). ISC, isochorismatase.
4. HHpred searching in PDB database (http://pfam.sanger.ac.uk/). ISC-hits, hits to known isochorismatase; ISCF-hits, hits to other members in isochorismatase family; OTH-hits, other family hits.
5. Signal peptide prediction was performed with the program SignalP 4.1 and iPSORT. Non-classically secreted proteins were predicted by SecretomeP 2.0 with threshold of neural network output scores 0.5 that is accepted by the algorithm. N, none leader sequence was identified. Y, the leader sequence was predicted.

Supplementary Table 3: Microbial strains and plant lines used in this study

No.	Name	Description and Purpose
		Phytophthora sojae
1	P6497	Race2; Wild-type (WT); Used as the transgenic recipient and a control.
2	T10	Transgenic line: WT+*PsIsc1*; Expression of *PsIsc1* is not altered; Used as a control.
3	ST6	Transgenic line: WT+*PsIsc1*; Expression of *PsIsc1* is silenced.
4	ST22	An independent transgenic line with same transgene as ST6.
5	OT3	Transgenic line: WT+*PsIsc1*; *PsIsc1* is over-expressed.
6	OT20	An independent transgenic line with same transgene as OT3.
7	GFP	Transgenic line: WT+*GFP*; Used as a control for protein secretion assay.
8	mRFP	Transgenic line: WT+*mRFP*; Used as a control for protein localization and virulent assay.
9	PsIsc1-mRFP	Transgenic line: WT+ *PsIsc1-mRFP*; The fusion of PsIsc1 and mRFP is expressed.
10	Avr1b-mRFP	Transgenic line: WT+ *Avr1b-mRFP*; The fusion of Avr1b and mRFP is expressed.
11	PsIsc1-Avr1bCt1	Transgenic line: WT+ *PsIsc1-Avr1bCt*; The fusion of Avr1b and C-terminal Avr1b (in which N-terminal host target signal is removed) is expressed.
12	PsIsc1-Avr1bCt3	An independent transgenic line with same transgene as PsIsc1-Avr1bCt1.
13	Avr1bCt-mRFP	Transgenic line: WT+ *Avr1bCt*; C-terminal Avr1b (in which the signal peptide and the N-terminal host target signal are removed) is expressed; Used as a negative control.
		Verticillium dahliae
1	V991	Wild-type (WT); A highly toxic defoliating WT isolate; Used as the transgenic recipient and a control.
2	Δ1-12	Deletion mutant: *VdIsc1* gene is deleted.
3	Δ1-18	An independent transgenic mutant that has the same deletion as Δ1-12.
4	Δ1-12VdIsc1	Complemented strain: *VdIsc1* gene is expressed in the deletion mutant (Δ1-12).
5	Δ1-12PsIsc1	Complemented strain: *PsIsc1* gene from *P. sojae* is expressed in the deletion mutant (Δ1-12).
6	Δ1-12VdIsc1^{A3}	Complemented strain: the mutated *VdIsc1* gene (substitution mutations of the three key amino acids) is expressed in the deletion mutant (Δ1-12).

7	Δ1-12PsIsc1^{A3}	Complemented strain: the mutated *PsIsc1* gene (substitution mutations of the three key amino acids) is expressed in the deletion mutant (Δ1-12).
8	Δ1-12VdIsc1$^{\Delta Nt}$	Complemented strain: *VdIsc1* gene lacking the N-terminal regions is expressed in the deletion mutant (Δ1-12).
9	Δ1-12PsIsc1$^{\Delta Nt}$	Complemented strain: *PsIsc1* gene lacking the N-terminal regions is expressed in the deletion mutant (Δ1-12).
		Phytophthora capsici
1	Strain 35	A *Phytophthora* pathogen that can infect *N. benthamiana*.
		Soybean
1	Williams	No *rps* gene; used as a control. *P. sojae* carrying *Avr1b* gene is virulent on this cultivar.
2	HARO13	Containing *Rps1b* gene. *P. sojae* carrying *Avr1b* gene is avirulent on this cultivar.
		Cotton
1	Lumian 21	A susceptible cotton cultivar.
		Nicotiana benthamiana
1	*N. benthamiana*	Used as a plant that can transiently express different exogenous genes.
		Arabidopsis thaliana
1	WT	Wild type Arabidopsis (Col2) that can be infected by *V. dahliae*.
2	*NahG* transformant	*Arabidopsis* expressing the *Pseudomonas putida NahG* gene in which SA levels are extremely low.

Supplementary Table 4: Oligonucleotides used in the study

No	Name	Sequences (5' to 3')a	Purpose
1	xhoI580F	caactcgagATACTTTCTGCTTTCGGCGTCA	amplify 5' flank sequence of *VdIsc1* gene
2	xhoI951R	gtactcgagCGAAAGGTTCCTTGTGTCATA	
3	Hind2207F	cataagcttGCTAAGCCGTGTAGTTCGCTAG	amplify 3' flank sequence of *VdIsc1* gene
4	Xba2678R	ctatctagaGTGCGTTGATCTGTGTGCCTTT	
5	JAGN617	ctgaagcttGGAGGTCAACACATCAATGCT	forward primer of *trpc* promoter
6	JAGN538	cctggatccTTAGGCGTAGTCAGGCACGTCGTAAGGATAGTTGATATCCTTGCTCGAGA	the reverse primer of *VdIsc1-HA*
7	TVDF	TCTACCCAAGCCTCGATCATGTCCTCATTCCGCTCC	fusion *trpc* promoter with *VdIsc1-HA*
8	TVDR	GGAGCGGAATGAGGACATGATCGAGGCTTGGGTAGA	
9	TVD-SPF	TCTACCCAAGCCTCGATCatgAGTGTACTCGTCATCATC	fusion *trpc* promoter with *VdIsc*$^{\Delta Nt}$*-HA*
10	TVD-SPR	GATGATGACGAGTACACTCATGATCGAGGCTTGGGTAGA	
11	TPS-SPF	CTACCCAAGCCTCGATCATGAGCGTGCTCTTCGTGTGC	fusion *trpc* promoter with *PsIsc1*$^{\Delta Nt}$*-HA*
12	TPS-SPR	GCACACGAAGAGCACGCTCATGATCGAGGCTTGGGTAG	
13	JAGN618	GAGCTCAGAGCCGCCATGATCGAGGCTTGGGTAG	fusion *trpc* promoter and *PsIsc1-HA*
14	JAGN619	CTACCCAAGCCTCGATCATGGCGGCTCTGAGCTC	
15	JAGN620	ACTGGATCCTTAGGCGTAGTCAGGCACGTCGTAAGGATAGATCCGCGCGCCCGTGTC	the reverse primer of *PsIsc1-HA*
16	5103mR1	GGCGTATTCGCCCTGGGCAGCGATGATGACGAGTACACTG	mutation of the 26th aspartic acid (D) of VdIsc1 to alanine (A)
17	5103mF1	GCCCAGGGCGAATACGCC	
18	5103mF2	AAGGCGAGGCTGTCGTGACGgctCACCACCCCGGTTCATTC	mutation of the 100th Lycine (K) of VdIsc1 to alanine (A)
19	5103mR2	CGTCACGACAGCCTCGCCTT	
20	5103mF3	GTCTCGACGACGGCCAGG	mutation of the 133th cysteine (C) of VdIsc1 to alanine (A)
21	5103mR3	CCTGGCCGTCGTCGAGACAGCGACGTGAGCCATGTACCC	
22	8159mR1	GCGGAAGACCTCCTGCACAGCGCACACGAAGAGCACGCT	mutation of the 25th aspartic acid (D) of PsIsc1 to alanine (A)

23	8159mF1	GTGCAGGAGGTCTTCCGC	
24	8159mR2	GTCGAAGACCTTGACGTC	mutation of the 90th Lycine (K) of PsIsc1 to alanine (A)
25	8159mF2	GACGTCAAGGTCTTCGACgctATGAAGTTCTCGATGCTC	
26	8159mR3	GAGGCACGTCTGCAGCACAGCCACGTGGGTCTCGATGCC	mutation of the 124th cysteine (C) of PsIsc1 to alanine (A)
27	8159mF3	GTGCTGCAGACGTGCCTC	
28	VdIsc1SF	gggATGTCCTCATTCCGCTCCA	for insertion of *VdIsc1* gene into pBinGFP2 vector
29	VdIsc1BaR	cgcggatccCTAGTTGATATCCTTGCTCG	
30	PsIsc1SF	gggATGGCGGCTCTGAGCTCG	for insertion of *PsIsc1* gene into pBinGFP2 vector
31	PsIsc1BaR	cgcggatccTTAGATCCGCGCGCCCGT	
32	GhPR1F	CTTGTCTCGTGGGGTTAGTC	qRT-PCR analysis of *PR1* gene in cotton
33	GhPR1R	TGTGAGCGTTGAGGTAGTCT	
34	Ghhistone3F	GGTGGTGTGAAGAAGCCTCAT	qRT-PCR analysis of cotton *histone3* gene
35	Ghhistone3R	AATTTCACGAACAAGCCTCTGGAA	
36	NbPR1F	GTGGACACTATACTCAGGTG	qRT-PCR analysis of *PR1* in *N. benthamiana*
37	NbPR1R	TCCAACTTGGAATCAAAGGG	
38	NbEF1a-QF	AGAGGCCCTCAGACAAAC	qRT-PCR analysis of EF1a in cotton
39	NbEF1a-QR	TAGGTCCAAAGGTCACAA	
40	VdActin-F1	CTGGTATCGTGCTTGACTCTG	qRT-PCR analysis of *V. dahliae Actin* gene (VDAG_08445.1)
41	VdActin-R1	GATTTCACGCTCGGCAGT	
42	VdIsc1F	GTTCATTCGCCGACACCA	qRT-PCR analysis of *V. dahliae VdIsc1* gene (VDAG_05103.1)
43	VdIsc1R	GGCAACGATGACATCCCAC	
44	JAGN452	ACAACAGACCACTGCGTCAACAC	qRT-PCR analysis of *V. dahliae VdIsc2* gene (VDAG_03530.1)
45	JAGN453	TCTTGGGTGTGTAGCCGTAGCAT	
46	JAGN450	TCGAGTCGCACGTTTGCATCAC	qRT-PCR analysis of *V. dahliae VdIsc3* gene (VDAG_06346.1)
47	JAGN451	TCTCCTTGACTATGCCGACGATGT	
48	actinAF2	ACTGCACCTTCCAGACCATC	Internal primers of *P. sojae actin* gene, for RT-PCR and real time PCR mRNA assays
49	actinAR2	CCACCACCTTGATCTTCATG	
50	pTOR-PsIsc1-EcoRI-F	ggaattcacaacaATGGCGGCTCTGAGCTC	For insertion of *PsIsc1* gene into *P. sojae* transformation vector
51	pTOR-PsIsc1-- XbaI-R	gctctagaTTAgtgatggtgatggtgatgGATCCGCGCGCCCGTGTC	
52	pTOR-PsIsc1-RFP-R1	GTCCTCGGAGGAGGCCATGATCCGCGCGCCCGT	For insertion of *PsIsc1* and *mRFP* fusion gene into *P. sojae*

53	pTOR-RFP-XbaI -R2	gctctagaTTAgtgatggtgatggtgatgGGCGCCGGTGGAGTGGCG	transformation vector
54	pTOR-PsIsc1-avr1b-R1	AGGTCAGTCACGCTGAAGGTGATCCGCGCGCCCGT	For insertion of *PsIsc1* and the C terminal of *Avr1b* fusion gene into *P. sojae* transformation vector
55	pTOR-avr1b-Xba I -R	gctctagaTCAGTGATGGTGATGGTGATG	
56	PsIsc1-RT-F	AGCGTGCTCTTCGTGTG	Internal primers of *PsIsc1*, used for RT-PCR assay for *PsIsc1* mRNA
57	PsIsc1-RT-R	TTGGTGCCGTGGATGAC	
58	PsIsc2/3-RT-F	CTACCTCCTGCCGATTCT	Internal primers of *PsIsc2/3*, used for RT-PCR assay for *PsIsc2/3* mRNA
59	PsIsc2/3-RT-R	CTTCTCCACCATTTCTTCAC	
60	pTORm-F	TTCACTCTCACGTGCCCAAG	Primers in pTOR promoter and pTOR terminator, for screening *P. sojae* transformants
61	pTORm-R	CTGCAACTTCGCACTCAGTG	
62	ACT20-F	TCGAGGCACCTAATTCTTGG	Internal primers of soybean *actin* gene, for RT-PCR and real time PCR mRNA assays
63	ACT20-R	GTGTCTGGATTGGTGGCTCT	
64	soybean-PR1 F	AACTATGCTCCCCCTGGCAACTATATTG	Internal primers of soybean *PR1*, used for RT-PCR assay for *PR1* mRNA
65	soybean-PR1 R	TCTGAAGTGGTAGCTTCTACATCGAAACAA	
66	Avr1bReF	ACCTTCAGCGTGACTGACCT	Internal primers of *Avr1b*, used for RT-PCR assay for *Avr1b* mRNA
67	Avr1bReR	GCGATTGCCAACCAGTTCT	
68	TOR-avr1bF	ggaattcacaacaATGCGTCTATCTTTTGTGC	For insertion of *Avr1b* and the *RFP* fusion gene into *P. sojae* transformation vector
69	TOR-avr1bR1	GTCCTCGGAGGAGGCCATGCTCTGATACCGGTGAAA	
70	TOR-RFPR2	gctctagaTTAGTGATGGTGATGGTGATGGGCGCCGGTGGAGTGGCG	
71	EntBF	ggaattccatATGGCTATTCCAAAATTAC	For insertion of *EntB* gene into prokaryotic expression vector pET21b
72	EntBR	ccgctcgagTTTCACCTCGCGGGAGAG	

73	VdIsc1F	cgcggatccATGTCCTCATTCCGCTCCA	For insertion of *VdIsc1* and *VdIsc1*^{A3} gene fusing His-tag into binary vector pBinGFP4
74	VdIsc1R	tgctctagactaatggtgatggtgatgatgGTTGATATCCTTGCTCGAG	
75	PsIsc1F	cgcggatccATGGCGGCTCTGAGCTCG	For insertion of *PsIsc1* and *PsIsc1*^{A3} gene fusing His-tag into binary vector pBinGFP4
76	PsIsc1R	tgctctagactaatggtgatggtgatgatgaATCCGCGCGCCCGTGTCGAA	
77	pTOR-Avr1b CtF	ggaattcacaacaATGACCTTCAGCGTGACTGACCTGT	For insertion of *Avr1b* C terminal and the *RFP* fusion gene into *P. sojae* transformation vector

Notes: Uppercase letters indicate bases that match the initial template. Lower case letters indicate mutations or 5’ extensions that do not match the initial template. Restriction sites introduced into the amplicon are underlined.

Supplementary Table 5: Constructs and methods used in the study

No	Plasmid Name	Sources	Construct and purpose
1	HKO.VdIsc1	pC1300HKO	Containing flanking sequences of *VdIsc1* gene for knocking out *VdIsc1* in *V. dahliae*
2	pC1300GN.VdIsc1	pC1300GN	Containing *VdIsc1* gene fused with *trpc* promoter for complement *VdIsc1* knock out mutant
3	pC1300GN. VdIsc1^{A3}	pC1300GN	Containing mutation of three key amino acid of VdIsc1 (D26A, K100A, C133A) (VdIsc1^{A3}) fused with *trpc* promoter for complement *VdIsc1* knock out mutant
4	pC1300GN. VdIsc1$^{\Delta Nt}$	pC1300GN	Containing deletion of 1-19 amino acid of VdIsc1 (VdIsc$^{\Delta Nt}$) fused with *trpc* promoter for complement *VdIsc1* knock out mutant
5	pC1300GN.PsIsc1	pC1300GN	Containing full length of *PsIsc1* gene fused with *trpc* promoter for complement *VdIsc1* knock out mutant
6	pC1300GN.PsIsc1^{A3}	pC1300GN	Containing mutation of three key amino acid of PsIsc1 (D25A, K90A, C124A) (PsIsc1^{A3}) fused with *trpc* promoter for complement *VdIsc1* knock out mutant.
7	pC1300GN.PsIsc1$^{\Delta Nt}$	pC1300GN	Containing deletion of 1-18 amino acid of PsIsc1 fused with *trpc* promoter for complement *VdIsc1* knock out mutant.
8	pBinGFP2.VdIsc1	pBinGFP2	Containing *PsIsc1* gene fused with the C-terminal of GFP for transient expression in *N. benthamiana*
9	pBinGFP2.PsIsc1	pBinGFP2	Containing *VdIsc1* gene fused with the C-terminal of GFP for transient expression in *N. benthamiana*
10	pBinGFP2.VdIsc1^{A3}	pBinGFP2	Containing mutation of three key amino acid of vdich1 (D26A, K100A, C133A) fused with the C-terminal of GFP for transient expression in *N. benthamiana*
11	pBinGFP2.PsIsc1^{A3}	pBinGFP2	Containing mutation of three key amino acid of psIsc1 (D25A, K90A, C124A) fused with the C-terminal of GFP for transient expression in *N. benthamiana*
12	pTOR.PsIsc1	pTOR	Containing *PsIsc1* gene fused with pTOR promoter, for overexpression and silence in *P. sojae.*
13	pTOR.PsIsc1-RFP	pTOR	Containing *PsIsc1* gene fused with *RFP*, for overexpression in *P. sojae.*
14	pTOR.PsIsc1-Avr1b	pTOR	Containing the C-terminal of Avr1b was fused to the C terminal of full length of PsIsc1, for

			overexpression in *P. sojae.*
15	pTOR.Avr1b-RFP	pTOR	Containing *Avr1b* gene fused with mRFP, for overexpression in *P. sojae.*
16	pTOR.Avr1bCt-RFP	pTOR	Containing *Avr1b* gene C terminal fused with RFP, for overexpression in *P. sojae.*
17	pET21b.EntB	pET21b	Containing *EntB* gene fused with His-Tag, for prokaryotic expression in *E. coli.*
18	pBinGFP4.VdIsc1	pBinGFP4	Containing *VdIsc1* gene fused with His-Tag, for transient expression in *N. benthamiana*
19	pBinGFP4.VdIsc1^{A3}	pBinGFP4	Containing *VdIsc1*^{A3} gene fused with His-Tag, for transient expression in *N. benthamiana*
20	pBinGFP4.PsIsc1	pBinGFP4	Containing *PsIsc1* gene fused with His-Tag, for transient expression in *N. benthamiana*
21	pBinGFP4.PsIsc1^{A3}	pBinGFP4	Containing *PsIsc1*^{A3} gene fused with His-Tag, for transient expression in *N. benthamiana*
